# Neuroretinal degeneration in a mouse model of systemic chronic immune activation observed by proteomics

**DOI:** 10.3389/fimmu.2024.1374617

**Published:** 2024-04-11

**Authors:** Asif Manzoor Khan, Maria Abildgaard Steffensen, Egle Paskeviciute, Ahmed Basim Abduljabar, Torben Lykke Sørensen, Henrik Vorum, Mogens Holst Nissen, Bent Honoré

**Affiliations:** ^1^ Department of Biomedicine, Aarhus University, Aarhus, Denmark; ^2^ Department of Immunology and Microbiology, University of Copenhagen, Copenhagen, Denmark; ^3^ Department of Ophthalmology, Zealand University Hospital, Roskilde, Denmark; ^4^ Faculty of Health and Medical Sciences, University of Copenhagen, Copenhagen, Denmark; ^5^ Department of Ophthalmology, Aalborg University Hospital, Aalborg, Denmark; ^6^ Department of Clinical Medicine, Aalborg University, Aalborg, Denmark

**Keywords:** LCMV, systemic immune activation, proteomics, biomarkers, mass spectrometry

## Abstract

Blindness or vision loss due to neuroretinal and photoreceptor degeneration affects millions of individuals worldwide. In numerous neurodegenerative diseases, including age-related macular degeneration, dysregulated immune response-mediated retinal degeneration has been found to play a critical role in the disease pathogenesis. To better understand the pathogenic mechanisms underlying the retinal degeneration, we used a mouse model of systemic immune activation where we infected mice with lymphocytic choriomeningitis virus (LCMV) clone 13. Here, we evaluated the effects of LCMV infection and present a comprehensive discovery-based proteomic investigation using tandem mass tag (TMT) labeling and high-resolution liquid chromatography–tandem mass spectrometry (LC-MS/MS). Changes in protein regulation in the posterior part of the eye, neuroretina, and RPE/choroid were compared to those in the spleen as a secondary lymphoid organ and to the kidney as a non-lymphoid but encapsulated organ at 1, 8, and 28 weeks of infection. Using bioinformatic tools, we found several proteins responsible for maintaining normal tissue homeostasis to be differentially regulated in the neuroretina and the RPE/choroid during the degenerative process. Additionally, in the organs we observed, several important protein pathways contributing to cellular homeostasis and tissue development were perturbed and associated with LCMV-mediated inflammation, promoting disease progression. Our findings suggest that the response to a systemic chronic infection differs between the neuroretina and the RPE/choroid, and the processes induced by chronic systemic infection in the RPE/choroid are not unlike those induced in non-immune-privileged organs such as the kidney and spleen. Overall, our data provide detailed insight into several molecular mechanisms of neuroretinal degeneration and highlight various novel protein pathways that further suggest that the posterior part of the eye is not an isolated immunological entity despite the existence of neuroretinal immune privilege.

## Introduction

Retinal degeneration resulting in complete or partial vision loss is estimated to affect more than 5.5 million individuals worldwide (1, 2). Age-related macular degeneration (AMD) is a retinal disease that affects the central vision of the eye due to retinal and photoreceptor damage in the macula. Growing evidence supports involvement of various immune-related proteins in disease progression. The steps leading to cellular degeneration appear to involve damage to the retinal pigment epithelium (RPE) layer that normally ensures retinal homeostasis. Furthermore, it has been reported that dying RPE cells and their debris may lead to increased photoreceptor death and vision loss (3). Although our understanding of the degenerative mechanisms has been enhanced, the investigation of the mechanisms by which retinal cells degenerate may still significantly improve our knowledge of AMD pathology. Some studies suggest the involvement of inflammatory components that may be systemic and present in peripheral tissues (4). In order to elucidate the underlying pathological mechanisms and search for such inflammatory components as well as to study the tissue response following the infection, we used a chronically infected mouse model (5) with the possibility of studying different organs comprehensively at different times. The neuroretina is an immune-privileged tissue (6), but studies showing a possible inflammatory component in the disease etiology suggest that some parts of the neuroretina may be more affected by an immune response than previously believed (7). RPE, being situated between the neuroretina and the choroid, forms a part of the blood–retina barrier (BRB) and provides crucial support to the retina; thus, diseases affecting the RPE also lead to increased vision loss (8). Based on these facts, we have studied these two entities separately. We have also analyzed reference organs without immune privilege for comparison: the spleen as a typical immunological organ with the possibility of following the immune reaction over time and the kidney as a non-immunological organ.

We used lymphocytic choriomeningitis virus (LCMV)-infected mice as a model system to investigate the effect of systemic infection on the retina. LCMV-infected mice have been widely used for the study of systemic immune activation (9–14). LCMV, an enveloped RNA virus and the prototypic arenavirus, provides a suitable model system for investigating the mechanisms of acute and chronic viral infection, host–cell interactions, and pathogenesis (12, 13, 15, 16). LCMV is a non-cytopathic virus and therefore any observed effects such as immune activation or degenerative processes reflect the host immune system responding to the presence of the virus and the attempt to eliminate it from the body (11, 15, 17). We used LCMV clone 13 as this persists in mice and induces a chronic infection that enables long-term analysis (5).

**Figure 1:**
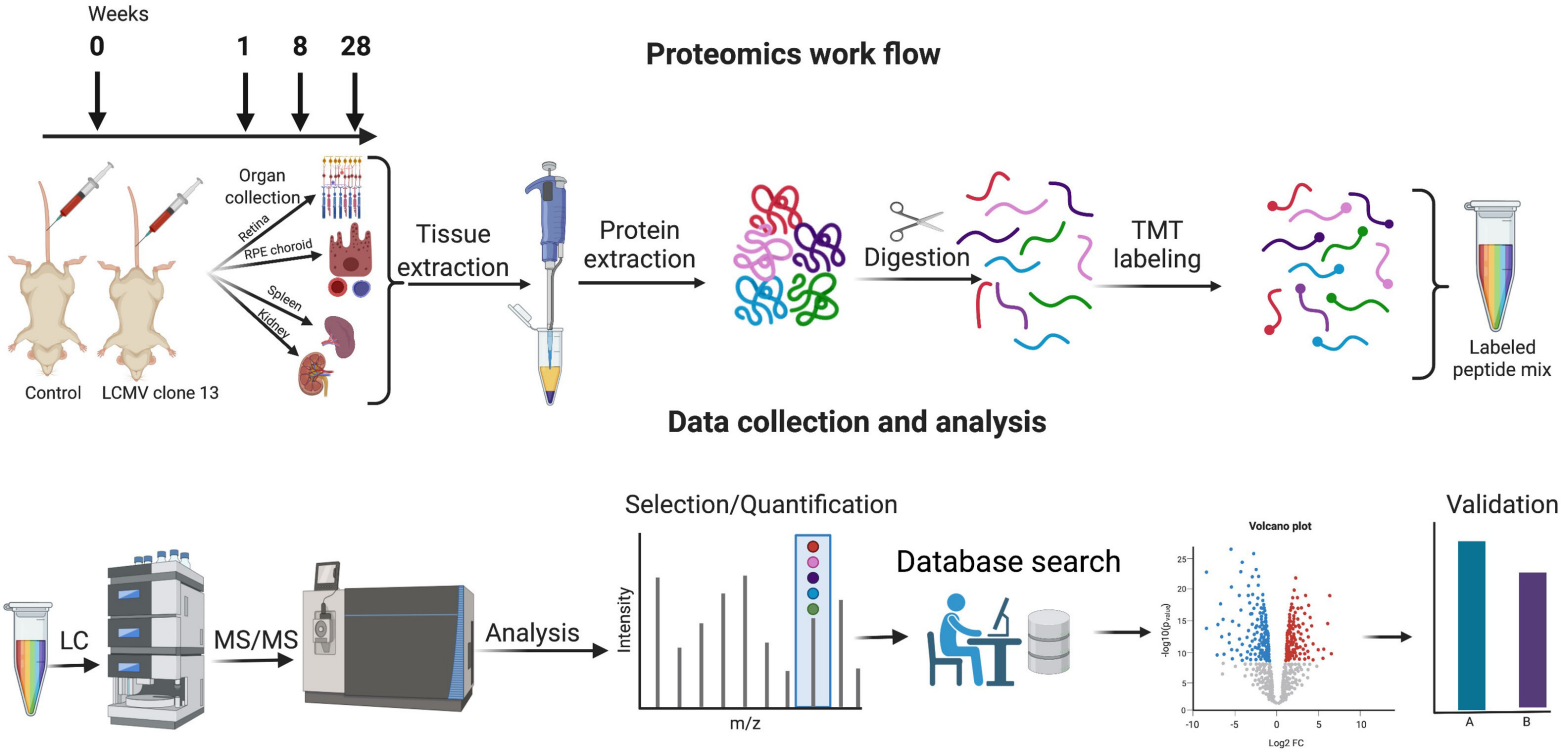
Proteomics workflow, data collection, and analysis. Mice were infected with LCMV clone 13 virus at day 0 and sacrificed at 1 week, 8 weeks, and 28 weeks. Retina, RPE/choroid, kidney, and spleen were harvested and subjected to proteomic analysis by TMT labeling and subsequent nano-liquid chromatography–tandem mass spectrometry. Validation of selected protein changes was performed by single reaction monitoring. Created with Biorender.com.

Encountering a pathogen brings changes in the expression of various proteins involved in the host response system. Proteomics is a highly suitable tool to study virus–host cell interactions because viral infection fundamentally affects host cell proteins (18). Using proteomics, we can gain an understanding of the perturbation of functional pathways that may affect cell signaling pathways, growth factor production, or apoptosis (19). Whole protein expression profiling has become a popular tool to identify proteins and their function in biological events. So far, the focus has been put on large-scale analyses of the host proteome, kinome, and transcriptome affected at the acute level of viral infection (20–23). On the other hand, not much has been done to unravel general mechanisms of the host cellular response occurring during a chronic infection on different organ proteomes. In the present study, we applied a proteomic analysis using tandem mass tag (TMT) labeling and high-resolution nano-liquid chromatography–tandem mass spectrometry (nLC-MS/MS) to comprehensively study the protein changes in each tissue in a time-dependent manner following the infection (**Figure 1**).

Our main aims were to identify and characterize differential expression of proteins, to detect enriched pathways in LCMV-infected mice, and to test whether any of the pathways were involved in degenerative processes.

## Materials and methods

### Animals

Female C57BL/6 wild-type (WT) mice were obtained from Taconic Farms (Ry, Denmark). All mice used in this study were 6–10 weeks old when experiments were initiated. They were housed in individually ventilated cages under specific pathogen-free conditions at the ALAAC-accredited animal facility at the Panum Institute (Copenhagen, Denmark). Mice coming from outside sources were allowed to rest for at least 1 week before entering an experiment. All procedures were approved by the national animal ethics committee (License: 2020-15-0201-00586) and were conducted in accordance with national Danish guidelines.

### LCMV infection model and harvest of tissues

LCMV clone 13 was originally obtained from M.B.A. Oldstone and amplified in-house (5). Mice were placed in a restrainer and infected with 2 × 10^6^ plaque-forming units (PFU) clone 13 in a tail vein. Naïve age-matched mice served as controls, and unless otherwise stated, all experimental groups contained five mice. On day 7 (1 week), day 56 (8 weeks), or day 196 (28 weeks) post-infection, mice were sacrificed by cervical dislocation and eyes, spleen, and kidneys were harvested in tubes and immediately placed on ice. Eyes were hemidissected posterior to the ora serrata, and the retinas were harvested in tubes with lysis buffer [5% SDS, 50 mM triethylammonium bicarbonate (TEAB), pH 8.5]. The RPE/choroid in the interior of the eyecups was scraped off with a 25G needle and collected in lysis buffer. Retinas and RPE/choroid from right and left eyes were pooled for further analysis. Kidneys and spleens were cut into small pieces and collected in lysis buffer. Kidneys from one mouse were pooled in a single tube. No tissues from different mice were pooled. All organs were stored at −80°C until further analysis.

### Experimental design

One week: LCMV clone 13 infected, sacrificed 7 days (1 week) after infection, RPE/choroid, retina, spleen, and kidney (short-term phase).

Eight weeks: LCMV clone 13 infected, sacrificed 56 days (8 weeks) after infection, retina, spleen, kidney, and RPE/choroid (intermediate phase).

Twenty-eight weeks: LCMV clone 13 infected, sacrificed 196 days (28 weeks) after infection, RPE/choroid, retina, spleen, and kidney (long-term phase).

### Protein extraction and digestion

Tissues were homogenized and sonicated in lysis buffer (5% SDS, 50 mM TEAB, pH 8.5), and the protein concentration was estimated by infrared spectrometry (Direct Detect Spectrometer, Merck KGaA, Darmstadt). Up to 100 µg was used for tryptic digestion with the suspension trapping method (24) using S-Trap™ spin columns (Protifi, Huntington, NY, USA) essentially as previously described (25). In short, proteins were reduced with Tris (2-carboxyethyl) phosphine hydrochloride (TCEP), alkylated with iodoacetamide, and acidified with a final phosphoric acid concentration of 1.2%. Seven volumes of S-Trap binding buffer (90% methanol, 100 mM TEAB) were added, applied to the filter unit, and washed. Digestion buffer (50 mM TEAB with trypsin) was added and incubated at 37°C overnight. Peptides were eluted stepwise in digestion buffer, 0.2% formic acid, and finally 0.2% formic acid and 50% acetonitrile. Elutions were pooled and dried, peptides were resuspended in TEAB, and the concentration was estimated by tryptophan fluorescence as previously described (26).

### Tandem mass tag labeling and fractionation of samples

Equal amounts of peptides from the five infected mice and the five control mice for each experiment were labeled with the TMT10plex™ Isobaric Mass Tagging kit (Thermo Fisher Scientific Inc., Waltham, MA, USA). Equal amounts of the 10 labeled samples were mixed and fractionated in eight samples using a Pierce™ High pH Reverse-Phase Peptide Fractionation Kit (Thermo Fisher Scientific). The peptide concentration in each fraction was measured and equal amounts of fractions, between 0.35 and 1 µg, were injected for each experiment. Two technical replicas were analyzed.

### Liquid chromatography–mass spectrometry

Analysis was performed on an LC-MS platform from Thermo Fisher Scientific Inc. (Waltham, MA, USA) consisting of a Dionex Ultimate 3000 RSLC nano-UPLC system coupled to an Orbitrap Fusion Tribrid mass spectrometer with an EasySpray ion source. Peptides were trapped on a µ-Precolumn (300 µm × 5 mm, C18, 2 mm, 100 Å) and separated using an analytical column (500 mm × 75 µm, PepMap RSCL, C18, 2 mm, 100 Å). Peptides were eluted by mixing buffer A (0.1% formic acid, 99.9% water) with buffer B (0.1% formic acid, 99.9% acetonitrile). The gradient was constructed by starting with 2% B. B was changed to 12% at 3 min, 25% at 149 min, 40% at 156 min, 80% at 159 min, 80% at 175 min, and 2% at 176 min continuing to 213 min. The mass spectrometer was operated in TMT synchronous precursor selection (SPS) MS^3^ mode. Full MS^1^ scans were obtained in the range 350–1500 m/z in the Orbitrap at a resolution of 120,000, an AGC target of 2 × 10^5^, and a maximum injection time of 50 ms. Precursor ions with charge states 2–7 were isolated with a quadrupole isolation window of 1.2 m/z, and MS^2^ acquisitions were performed in the linear ion trap in auto scan range mode and applying a collision-induced dissociation (CID) with an energy of 35%, an AGC target of 2 × 10^4^, and a maximum injection time of 70 ms. MS^3^ scans were performed with SPS, a quadrupole isolation window of 2 m/z, high-energy collisional dissociation (HCD) with an energy of 65%, an orbitrap resolution of 50,000, a scan range of 100–500 m/z, an AGC target of 3 × 10^4^, and a maximum injection time of 110 ms.

### Proteomic data analysis

Mass spectrometry data were processed further with MaxQuant version 1.6.6.0 (Max Planck Institute of Biochemistry, Martinsried, Germany) (27) and further processed in Perseus versions 1.6.2.1, 1.6.6.0, and 1.6.14.0 (Max Planck Institute of Biochemistry, Martinsried, Germany) (28). Data were filtered in Perseus for proteins only identified by post-translational modification, identified in the reverse database, or identified as contaminants. Reporter ion intensities were log_2_ transformed. At least two unique peptides were required for identification and 100% valid values in total were required. Finally, the means of the two replicas were used for calculations. Only protein changes with *p*-values <0.05 were considered to be significantly changed. The mass spectrometry proteomics data have been deposited to the ProteomeXchange Consortium (http://proteomecentral.proteomexchange.org) via the PRIDE partner repository (29) with the dataset identifier PXD050187.

### Bioinformatic analysis

Data analysis of the differentially regulated protein networks has been described previously (30). To decipher the role of significantly regulated proteins, we used Ingenuity Pathway Analysis (IPA) (Qiagen Inc., Hilden, Germany, https://www.qiagenbioinformatics.com/products/ingenuity-pathway-analysis). The algorithms used for IPA is described by Krämer (31). Bioinformatic analysis was also performed using Cytoscape plug-ins (CluePedia, ClueGO) with default parameters to find the biological networks and interrelations of the functional groups as described previously (32, 33).

### Targeted proteomics with selected reaction monitoring

Selected reaction monitoring (SRM) was performed essentially as described previously (34). In short, tryptic peptides were prepared with S-Trap™ spin columns and 1 µg of each sample was injected in the LC-MS system. This system consisted of a Dionex Ultimate 3000 RSLC nano-UPLC coupled to a TSQ Quantiva from Thermo Fisher Scientific Inc. FASTA files for each of the proteins analyzed were downloaded from UniProt and entered into Skyline (35, 36) and used to prepare a method. The peak intensities were analyzed using Skyline and used to compare LCMV-infected samples with control samples.

## Results

### Protein expression changes in each tissue

**Figure 2:**
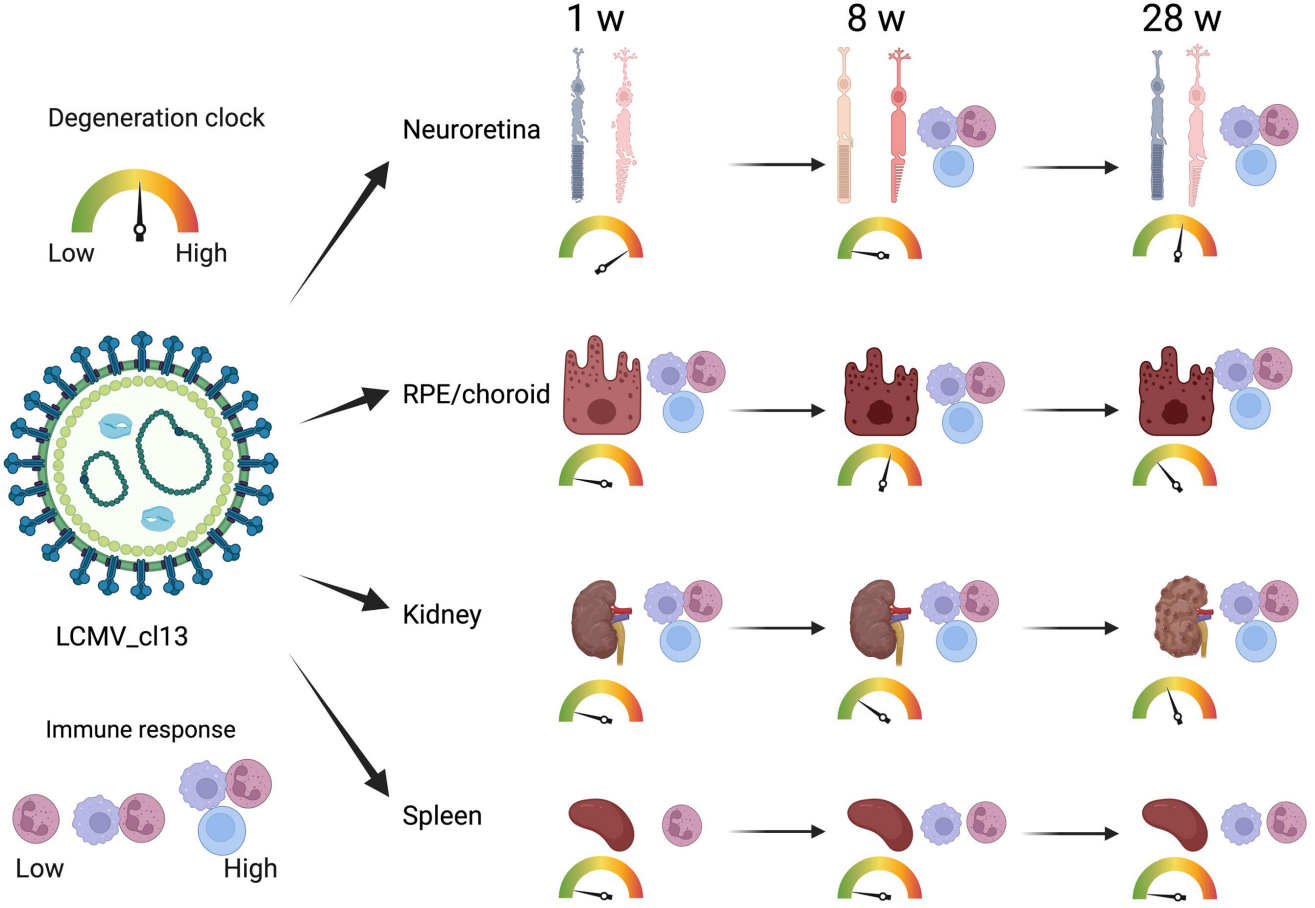
Time-dependent immune response and degeneration in various organs. LCMV infection induced various degrees of time-dependent immune response and degeneration in the retina, RPE choroid, and kidney. In the spleen, the immune response did not result in degeneration. Created with Biorender.com.

**Figure 3:**
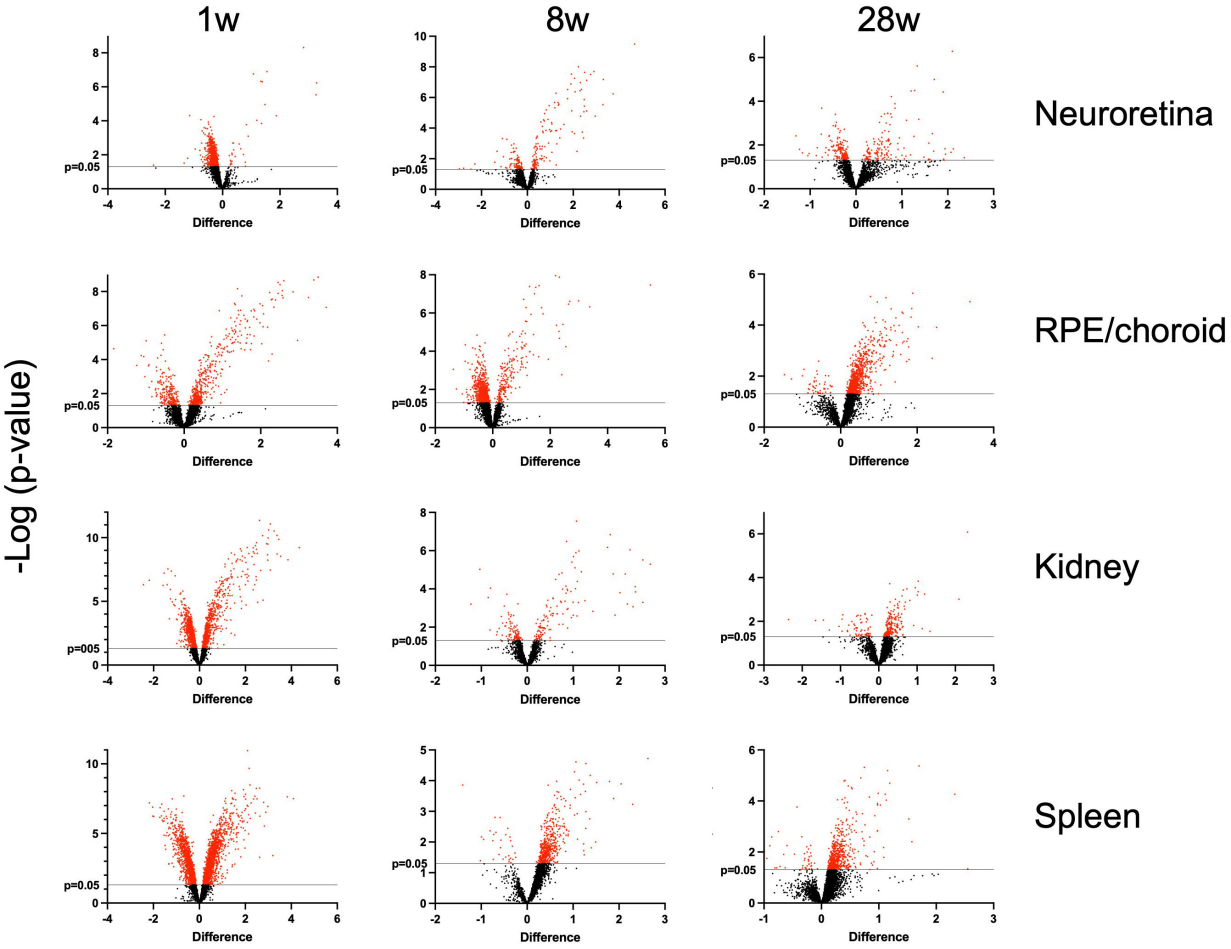
Volcano plots. Indicated on the *x*-axis are log_2_ fold-changes of LCMV-infected mice (*n* = 5) versus age-matched control mice (*n* = 5). On the *y*-axis, -log_10_(p-values) obtained with a *t*-test are shown. Vertical lines show *p* = 0.05. Red spots show the significantly differentially expressed proteins. 1 w, 1 week of infection; 8 w, 8 weeks of infection; and 28 w, 28 weeks of infection. The number of proteins in each of the tissues at the given time is given in **Table 1**.

In this study, we investigated the proteins and degeneration that take place in four different tissues—neuroretina, RPE/choroid, kidney, and spleen—in mice infected with LCMV clone 13, at 1, 8, and 28 weeks of infection (**Figure 2**). The effects of the infection on the proteomes varied substantially between the four different tissues. The mean number of identified proteins in the tissues was 4,287 (range: 3,261–4,948) as seen in **Table 1**. By comparing the LCMV-infected tissue with age-matched naïve tissue, we obtained the number of differentially expressed proteins as given in **Table 1** and shown in the volcano plots in **Figure 3** where significantly differentially expressed proteins are indicated above the horizontal lines (*p* < 0.05). Generally, the LCMV infection changed the expression level of various proteins massively in each tissue, especially after 1 week of infection. The fraction of the identified proteins that changed expression level was 28% in the neuroretina, 17% in RPE/choroid, 38% in kidney, and as much as 55% in spleen. At 8 to 28 weeks, we found a marked decrease in the fraction of differentially expressed proteins to between 5% and 13% in the neuroretina, kidney, and spleen. Only the RPE/choroid deviated from this as the amount of differentially expressed proteins remained relatively stable at approximately 17%–22% in the period from 1 week to 28 weeks. The complete list of identified and differentially expressed proteins in each tissue is provided as supplementary information (**Supplementary Tables S1**–**S4**). A set of proteins were also analyzed by targeted proteomics using SRM for verification of selected protein changes observed by the discovery-based technique (**Supplementary Figure S1**). Generally, the protein changes observed by the discovery technique were confirmed by SRM underlining the validity of the results.

**Table 1:** Number of identified and differentially expressed proteins in each tissue.

	Neuroretina			RPE/choroid			Kidney			Spleen		
	ID	Differentially expressed	%	ID	Differentially expressed	%	ID	Differentially expressed	%	ID	Differentially expressed	%
1 week	4670	1,288	28	4434	743	17	4198	1,600	38	4636	2,547	55
8 weeks	4948	240	5	3876	869	22	4039	236	6	4671	516	11
28 weeks	4049	311	8	4082	868	21	3261	279	9	4592	613	13

To study the biological changes induced by the infection, we used two bioinformatic tools, IPA and Cytoscape. As a first approach, IPA constructs a summary of the most significant entities observed at various times in each organ based on machine learning technique. The summary is shown in **Supplementary Figure S2**; the significantly perturbed “Canonical pathways” in each organ are listed in **Supplementary Tables S5**–**S8**, and the significant changes for “Diseases & Functions” are shown in **Supplementary Tables S9**–**S12**. A detailed description of the proteomic changes in each tissue at various times may be found in the **Supplementary Materials**. Here, we will present the major observations.

### Biological changes in the neuroretina and RPE/choroid as a function of infection time

#### Retinal function

The neuroretina and RPE/choroid are instrumental for the function of the eye. This function was affected differently in the two compartments. In the neuroretina, early in the infection at 1 week, we observed several degenerative processes that involved the retina, the eye, or photoreceptors. **Tables 2**–**5** list the disease or function annotation obtained with the IPA for the neuroretina and for RPE/choroid. For the neuroretina at 1 week of infection, **Table 2** lists 48 annotations containing one of the words—retina, eye, or photoreceptor—that were significantly perturbed in the analysis (*p* < 0.05). Moreover, nine of them were significantly increased (*z*-score ≥2) or decreased (*z*-score ≤−2). For example, “Retinal degeneration”, “Degeneration of eye”, and “Degeneration of photoreceptors” were significantly increased while “Function of retina”, “Quantity of photoreceptors”, “Function of retinal cells”, “Quantity of retinal cells”, “Function of photoreceptors”, and “Response of photoreceptors” were all significantly decreased (**Table 2**). This strong loss of neuroretinal function was not seen later at approximately 8 weeks of infection. In fact, at 8 weeks of infection, none of the significantly perturbed diseases or functions contain the words retina, eye, or photoreceptor in the annotations (see **Supplementary Table S5A** for details). Later, however, at 28 weeks of infection, the inhibition of several neuroretinal functions was again apparent as 39 annotations contain either retina, eye, or photoreceptor (**Table 3**). Three diseases or functions were significantly increased, and one was significantly decreased (**Table 3**). In RPE/choroid, early in the infection at 1 week, we observed no signs of degenerative processes. We could not detect perturbations with annotations that include retina, eye, or photoreceptor (see **Supplementary Table S6A** for details). However, substantial degenerative processes were observed later in the infection at 8 weeks (**Table 4**). **Table 4** lists 23 significant perturbations with annotations that include retina, eye, or photoreceptor and with 4 of these perturbations also significantly increased or decreased. All of them indicate functional loss in RPE/choroid. At 28 weeks of infection, there was still a sign of degeneration with significant perturbation of the annotation “Morphology of photoreceptors” (**Table 5**).

**Table 2:** Neuroretina and disease or function annotations at 1 week of LCMV infection.

Diseases or function annotation	*p*-value	Predicted activation state	Activation *z*-score	No. of molecules
Progressive retinal atrophy	1.89E-06			47
Hereditary retinal degeneration	1.59E-05			60
Retinal dystrophy	1.68E-05			51
Morphology of eye cells	3.22E-05			40
Hereditary retinal disease	3.93E-05			63
Retinal degeneration	3.94E-05	Increased	4.943	81
Degeneration of eye	3.95E-05	Increased	4.943	82
Morphology of retinal cells	4.66E-05			37
Abnormal morphology of neurosensory retina	5.39E-05			26
Morphology of retina	8.61E-05			55
Autosomal recessive retinal degeneration	9.02E-05			40
Electrophysiology of retinal rods	1.09E-04			23
Morphology of photoreceptors	1.58E-04			32
Formation of eye	2.67E-04		−1.980	84
Autosomal recessive retinal dystrophy	3.45E-04			18
Function of retina	4.43E-04	Decreased	−3.092	23
Electrophysiology of eye	6.65E-04			33
Abnormal morphology of retinal layer	7.79E-04			24
Morphology of eye	8.31E-04			65
Morphology of photoreceptor outer segments	8.84E-04			25
Hereditary eye disease	1.09E-03			99
Abnormal morphology of retina	1.29E-03			46
Quantity of photoreceptors	1.42E-03	Decreased	−4.023	20
Development of retina	1.62E-03		−1.980	24
Abnormal morphology of photoreceptors	1.62E-03			28
Cone photoreceptor disorder	1.64E-03			21
Function of retinal cells	2.11E-03	Decreased	−2.595	20
Quantity of retinal cells	2.39E-03	Decreased	−3.777	21
Degeneration of photoreceptors	2.78E-03	Increased	3.470	27
Formation of retinal cells	3.06E-03			20
Development of photoreceptors	3.94E-03			19
Morphogenesis of eye	4.98E-03			15
Abnormal morphology of photoreceptor outer segments	5.09E-03			22
Autosomal dominant retinal dystrophy	5.27E-03			11
Scotopic b-wave response of retina	5.80E-03		−1.067	4
Morphology of retinal cone cells	6.40E-03			7
Abnormal morphology of photoreceptor inner segments	6.70E-03			10
Morphogenesis of photoreceptors	7.39E-03			5
Abnormal pigmentation of retina	7.39E-03			5
Function of photoreceptors	8.49E-03	Decreased	−2.595	9
Abnormal morphology of retinal pigment epithelium	8.82E-03			11
Abnormal morphology of eye	9.45E-03			52
Electrophysiology of retinal cone cells	1.08E-02			12
Response of photoreceptors	1.66E-02	Decreased	−2.213	6
Abnormal morphology of retinal cone cells	1.66E-02			6
Abnormal electrophysiology of eye	1.91E-02			20
Abnormal morphology of retinal ganglion cells	2.00E-02			5
Macular dystrophy 2, bull’s eye	2.10E-02			3

**Table 3:** Neuroretina and disease or function annotations at 28 weeks of LCMV infection.

Disease or function annotation	*p*-value	Predicted activation state	Activation *z*-score	No. of molecules	
Electrophysiology of eye	2.86E-10			23
Electrophysiology of retinal rods	1.29E-08			16
Degeneration of photoreceptors	6.41E-08	Increased	2.663	16
Abnormal morphology of retina	1.69E-07			25
Degeneration of eye	1.71E-07	Increased	3.215	33
Formation of eye	3.23E-07		−1.353	33
Retinal degeneration	4.17E-07	Increased	3.409	33
Morphology of retina	4.23E-07			26
Morphology of eye cells	5.78E-07			19
Abnormal morphology of eye	5.94E-07			28
Morphology of retinal cells	8.20E-07			18
Morphology of eye	1.55E-06			30
Morphology of photoreceptors	2.71E-06			16
Abnormal morphology of photoreceptors	4.91E-06			15
Abnormal electrophysiology of eye	1.21E-05			13
Function of retina	1.61E-05		−1.673	12
Hereditary retinal degeneration	4.27E-05			21
Abnormal morphology of photoreceptor outer segments	6.40E-05			12
Progressive retinal atrophy	1.02E-04			16
Cell death of eye cells	1.23E-04		0.426	10
Abnormal function of eye	1.44E-04			8
Function of retinal cells	1.63E-04		−1.951	10
Quantity of photoreceptors	1.63E-04	Decreased	−2.615	10
Retinal dystrophy	1.76E-04			17
Function of photoreceptors	2.20E-04		−1.951	6
Autosomal dominant retinal dystrophy	2.34E-04			7
Formation of photoreceptor outer segments	2.61E-04			5
Length of photoreceptor segment	2.61E-04			5
Abnormal morphology of retinal rods	2.61E-04			5
Cell death of retinal cells	3.88E-04		1.134	9
Development of photoreceptors	3.88E-04			9
Recovery of retinal cells	4.59E-04			3
Light sensitivity of photoreceptors	4.59E-04			3
Electrophysiology of retinal cone cells	6.99E-04			7
Apoptosis of eye cells	6.99E-04		−1.109	7
Degeneration of retinal rods	8.41E-04		0.958	5
Early-onset retinal dystrophy	1.02E-03			4
Autosomal recessive retinal degeneration	1.14E-03			13
Autosomal dominant retinal degeneration	1.19E-03			8

**Table 4:** RPE/choroid and disease or function annotations at 8 weeks of LCMV infection.

Disease or function annotation	*p*-value	Predicted activation state	Activation *z*-score	No. of molecules
Electrophysiology of eye	1.56E-08			30
Formation of eye	1.63E-08		−0.332	80
Degeneration of eye	3.64E-08	Increased	4.022	68
Retinal degeneration	5.26E-08	Increased	4.022	67
Abnormal morphology of retina	9.26E-08			42
Abnormal morphology of eye	1.62E-07			56
Morphology of eye	6.06E-07			61
Morphology of retina	8.33E-07			44
Electrophysiology of retinal rods	2.10E-06			18
Morphology of eye cells	1.04E-05			30
Degeneration of photoreceptors	1.32E-05	Increased	2.763	24
Morphology of retinal cells	1.52E-05			27
Function of retina	2.40E-05	Decreased	−2.400	17
Function of retinal cells	5.04E-05		−1.951	15
Hereditary retinal disease	5.07E-05			41
Hereditary retinal degeneration	6.03E-05			39
Morphology of photoreceptors	7.42E-05			24
Development of retina	1.31E-04			17
Abnormal morphology of photoreceptors	1.63E-04			22
Progressive retinal atrophy	2.68E-04			27
Maintenance of photoreceptors	2.89E-04			10
Abnormal electrophysiology of eye	3.21E-04			16
Neovascularization of eye	3.54E-04		0.750	18

**Table 5:** RPE/choroid and disease or function annotations at 28 weeks of LCMV infection.

Disease or function annotation	*p*-value	Predicted activation state	Activation *z*-score	No. of molecules
Morphology of photoreceptors	8.28E-04			19

**Figure 4:**
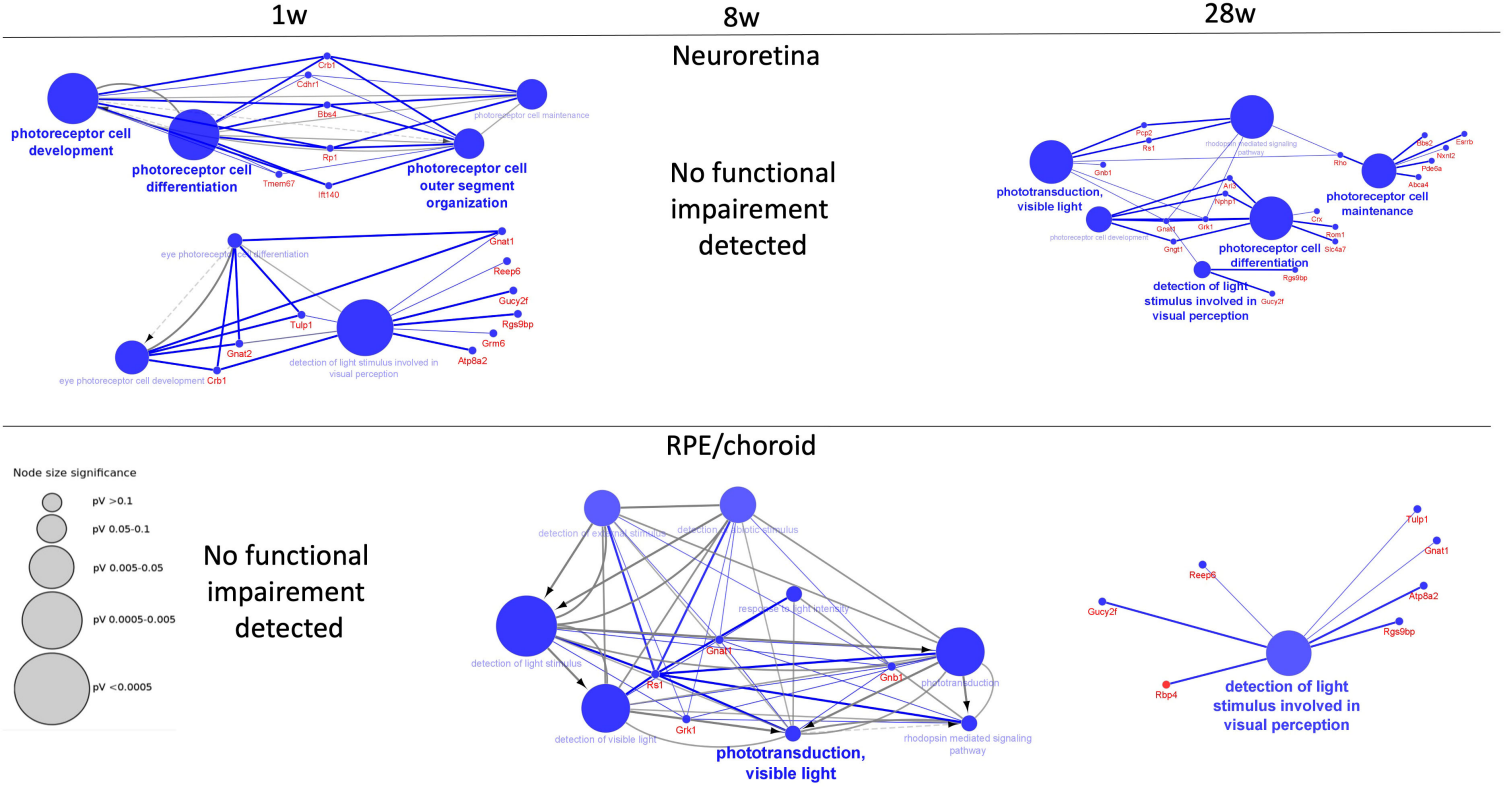
Decreased functions of neuroretina and RPE/choroid. Functions of neuroretina and RPE/choroid versus time after the start of infection. Examples of significantly perturbed and decreased functions of the retina and RPE/choroid, analyzed in Cytoscape. Large blue nodes with blue text reveals decreased functions. Node size indicates significance as given by the size of the gray nodes. Proteins that participate in the functions are given with red names. In neuroretina, a strong inhibition of neuroretinal function was seen early at 1 week (1 w) of infection with improvement at 8 weeks (8 w) where no functional impairment was detected. Decreased function was also observed late at 28 weeks (28 w) of infection. In the RPE/choroid, no early decreased retinal function was observed. Decreased functions were observed at 8 weeks and at 28 weeks.

This time-dependent functional loss was also observed by a specific canonical pathway for the eye function “Phototransduction pathway”, which was significantly perturbed in the neuroretina at 1 week, not at 8 weeks but again at 28 weeks of infection, while in RPE/choroid, it was not significantly perturbed at 1 week, but at 8 weeks and 28 weeks of infection (**Supplementary Tables S5A–C**, **S6A–C**). Thus, a functional loss was observed in the neuroretina at 1 week with improvement at 8 weeks and with reappearance of a functional loss at 28 weeks while no functional loss was observed in the RPE/choroid early at 1 week but at 8 weeks and, to some extent, at 28 weeks. Examples of the time-dependent functional loss in the neuroretina and RPE/choroid are shown in **Figure 4**. For details, see **Supplementary Tables**.

#### Immune response activation

The proteomic changes that indicated activation of the immune response occurring in the neuroretina and in RPE/choroid were also very different in the two compartments. In the neuroretina, the observed protein changes showed no strong sign of activation of an immune response early in the infection at 1 week. Only 17 of the 1,288 proteins that significantly changed expression level could be explained to change as a response to exposure of the neuroretina to a single cytokine, IFNB1 (**Supplementary Figure S2A**, 1 week). Moreover, only one immune pathway, “B cell development”, was significantly perturbed (**Supplementary Table S5A**). Protein changes that indicated signs of an antibody response (**Supplementary Figure S3A**) or complement activation (**Supplementary Figure S3B**) in the neuroretina could not be detected either as significantly perturbed at 1 week. Together, this indicated that there was only a slight immune response in the neuroretina at 1 week. However, later in the infection, we detected stronger signs of immune response in the neuroretina. Thus, at 8 and 28 weeks, more than approximately a third to a half of the significantly changed proteins (>100 protein changes) could be explained by changes of cytokine exposure (**Supplementary Figure S2A**, 8 weeks) and several immune response pathways were observed to be significantly perturbed and significantly stimulated in the neuroretina. Examples of this at 8 weeks were the diseases or functions “Immune response of cells” (**Supplementary Table S9B**), “Leukocyte migration” (**Supplementary Table S9B**), “Migration of phagocytes” (**Supplementary Table S9B**), and the canonical pathway “Th1 pathway” (**Supplementary Table S5B**). In the neuroretina at 8 weeks, the perturbed immune pathways included indication of B-cell signaling (**Supplementary Table S5B**) and antibody response (**Supplementary Table S9B**). This was most pronounced at 8 weeks of infection. At 28 weeks of infection, “B cell development” was significantly perturbed in neuroretina (**Supplementary Table S9C**).

RPE cells play an important role in the retinal immune homeostasis (37). In RPE/choroid, a strong immune response was seen already at the beginning of the infection at 1 week and throughout the observation period to 28 weeks of infection. Thus, more than a third of the significantly changed proteins (>300 protein changes) could be explained by changes to cytokine exposure (**Supplementary Figure S2B**) and several immune functions were significantly perturbed and significantly stimulated (**Supplementary Tables S10A–C**). Details of the specific and significant immune reactions observed are available in the **Supplementary Materials**.

#### Other major biological changes

In the neuroretina at 1 week of infection, we generally observed inhibition of other biological functions as well, e.g., cell cycle control of chromosomal replication that was strongly inhibited (**Supplementary Table S5A**). We further found a downregulation of the functional assembly of the RNA polymerase complex (**Supplementary Table S5A**), which leads to downregulation of the transcription and thereby downregulation of the protein synthesis. Also, metabolic pathways like triglyceride synthesis were inhibited (**Supplementary Table S5A**). A similar significant inhibition was not seen in the RPE/choroid at 1 week. Indeed, one of the observed functions, cell cycle control of chromosomal replication, was even stimulated in the RPE/choroid at 1 week (**Supplementary Table S6A**) while it was inhibited in the neuroretina (**Supplementary Table S5A**). Other examples with differences between the compartments were the signaling between the cytoskeleton intracellularly with the extracellular matrix. This seemed to be affected in the RPE/choroid throughout the whole period, increased at 1 week, decreased at 8 weeks, and again increased at 28 weeks (**Supplementary Figure S2B**). Integrins are part of this signaling process, and it has previously been reported that LCMV-mediated inhibition of the integrin pathway is an immune evasion mechanism to promote its virulence and pathogenesis (38). Apparently, the inhibition was overcome at the late stage of infection at 28 weeks. This function was not significantly perturbed in the neuroretina. Endoplasmic reticulum (ER) stress pathway was also found to be enhanced due to chronic LCMV infection in the RPE/choroid at 28 weeks of infection (**Supplementary Table S6C**). It has previously been reported that virus-mediated infection activates ER stress to facilitate viral survival and its replication (39).

### Biological changes in kidney as a function of infection time

#### Kidney function

Degeneration was not a problem initially at 1 week of infection; in fact, some kidney failure and impairment functions were significantly inhibited (**Supplementary Table S11A**). Some other degenerative processes in the kidney became apparent from 8 weeks of infection with a significant perturbation of renal impairment (**Supplementary Table S11B**) and later at 28 weeks where the function end-stage renal disease was significantly perturbed (**Supplementary Table S11C**).

#### Immune response activation

In the kidney, we observed a strong response early in the infection with changes in almost 500 proteins, which could be explained by changes in cytokine exposure (**Supplementary Figure S2C**, 1 week). This effect decreased to 117 proteins at 8 weeks and 86 proteins at 28 weeks (**Supplementary Figure S2C**, 8 and 28 weeks). The immune response was strong during the whole period based on the observation of several pathways, which were significantly stimulated and an indication of substantial decrease in the susceptibility to infection, especially at 1 to 8 weeks in the period (**Supplementary Figure S2C**).

#### Major biological changes

**Figure 5:**
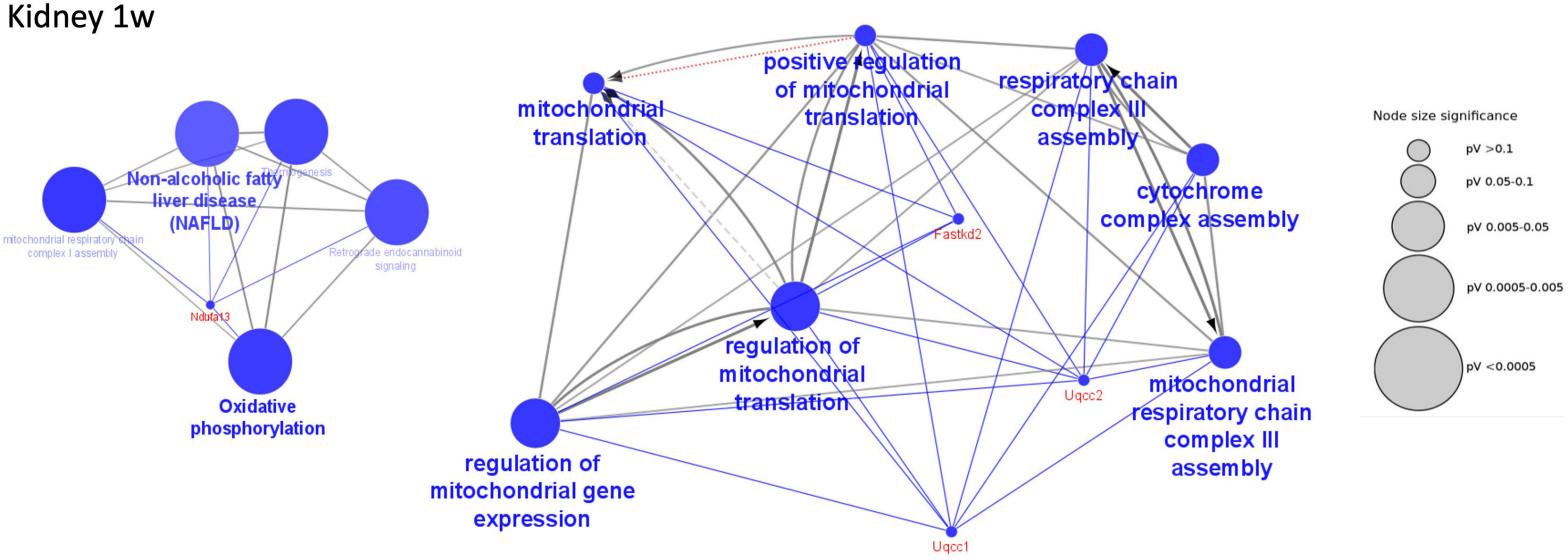
Decreased functions in kidney at 1 week of infection. Examples of significantly perturbed and decreased functions of the kidney at 1 week of infection, analyzed in Cytoscape. Large blue nodes with blue text reveal decreased functions. Node size indicates significance as given by the size of the gray nodes. Proteins that participate in the functions are given red names. Examples of mitochondrial functions in the kidney that were decreased at 1 week of infection.

A main observation was a strong mitochondrial dysfunction at 1 week including significant inhibition of acetyl-CoA synthesis, TCA cycle, and oxidative phosphorylation affecting the ATP generation (**Figure 5** and **Supplementary Table S11A**). For example, the mitochondrial protein UQCC2 and its binding partner UQCC1, which both were downregulated at 1 week, are required for maintaining the mitochondrial complex (40). Mitochondrial dysfunction has been described in various severe and chronic disorders (40–42). The mitochondrial inhibition was relieved at the later stages of infection at 8 to 28 weeks. Another pathway found to be upregulated at 1 week of LCMV infection was “NRF2-mediated oxidative stress response” (**Supplementary Table S7A**). This signaling pathway has been reported in controlling redox homeostasis and antiviral/inflammatory responses to infection (43). This finding goes hand in hand with our finding of increased immune activity in the first week of LCMV-mediated infection.

### Biological changes in spleen as a function of infection time

#### Spleen function

No major degenerative processes were detected throughout the period.

#### Immune response activation

In the spleen, we observed a strong response to cytokines early in the infection at 1 week with changes in almost 500 proteins, which could be explained by changes in cytokine exposure (**Supplementary Figure S2D**). The effect decreased to a moderate response at 8 weeks, while at 28 weeks, no changes could be seen as a response to cytokines (**Supplementary Figure S2D**). However, at 28 weeks of infection, approximately 150 protein changes could be explained by changes of exposure to growth factors (**Supplementary Figure S2D**). Surprisingly, the immune response was weak at 1 week as several immune functions were significantly inhibited but was apparent later at 8 weeks of infection and was also observed at 28 weeks of infection (see supplementary description of bioinformatics). The pattern with a weak immune activity in the early phase and stronger in the late phase may suggest that the immune mechanisms may initially have been compromised. As an example, it has been reported that silver nanoparticle-induced neurotoxicity may also cause various cellular responses in a tightly regulated, time-dependent manner (30).

#### Major biological changes

Some major biological changes were seen, e.g., the gene expression was affected initially, at 1 week, by inhibition of the translation while transcription was stimulated later at 28 weeks (**Supplementary Figure S2D**). The communication between the extracellular matrix and the cytoskeleton through integrins was affected, being inhibited at the early stage of infection, and increased at the late stage with increased formation of the cytoskeleton (**Supplementary Figure S2D**). The oxidative phosphorylation was stimulated at 8 weeks of infection (**Supplementary Table S8B**; **Supplementary Figure S2D**).

## Discussion

Retinal degeneration is the main cause of vision loss, which affects a vast population worldwide and poses a huge burden on society. Dysregulation of the immune system plays a crucial role in the pathogenesis of various neurodegenerative conditions including the retina (44). The purpose of this study was to explore and identify novel protein pathways and immune signaling mechanisms that are involved in retinal degeneration and photoreceptor cell loss.

The retina and RPE/choroid are instrumental for the visual function of the eye. This function was differently affected in the two compartments. In the neuroretina, early in the infection at 1 week, several degenerative processes involving the retina, the eye, or photoreceptors were observed to be significantly increased. This massive downregulation of proteins involved in photoreceptor function was not observed later at approximately 8 weeks of infection, and we did not find any apparent signs of functional inhibition. Later, however, at 28 weeks of infection, the inhibition of several neuroretinal functions was again apparent. In the neuroretina, the low immune response in 1 week of LCMV infection and high immune activity in the RPE cells can be explained by the retina as tightly regulated and protected by the BRB. The RPE layer next to the neuroretina constitutes the outer blood–retina barrier (oBRB) integrity (45). The viral infection may have affected the neuroretina in the first week directly or indirectly with substantial loss of function. This seemed to be an overall reversible process as the neuroretinal function improved again at 8 weeks of infection. Later, however, at 28 weeks of infection, functional loss was again apparent, which may be explained by a steadily increased dysfunctional retinal barrier due to long viral exposure. Retinal homeostasis is dependent on RPE/choroid. Thus, the initial functional loss in the neuroretina is apparently reversible and may be improved by a well-functioning RPE/choroid, while in the late phase, the defective RPE/choroid is not able to fully support the retinal function. Moreover, we found complement pathways to be slightly activated in the neuroretina at 8 and 28 weeks. It has been suggested that that complement will only be activated in the retina when the retinal barrier is broken (46). Impaired retinal barrier integrity results in many retinal diseases including AMD and diabetic retinopathy (7) and complement activation is known to be involved in the pathogenesis of retinal disease and retinal aging (47, 48). Taken together, these results suggest that a dysfunctional retinal barrier and inflammation is actively present in the later stages of LCMV-mediated infection in the mouse model.

In RPE/choroid, degenerative processes were not seen early in the infection but became apparent from 8 weeks and throughout the period. The protein changes observed in response to cytokines were generally much more pronounced in the RPE/choroid than in neuroretina. The high cytokine effect on the RPE/choroid proteome but not on the neuroretina early in the infection can be due to the BRB activity where RPE cells play an important role in insulating the retina (7). Studies on various *in vivo* models have shown that cumulative effects of innate immune cell influx, altered immune signaling, inflamed environment, and reduced tissue homeostasis lead to RPE-mediated retinal degeneration. Overall, this contributes to acceleration of disease severity (49, 50).

## Conclusions

In summary, time-course quantitative proteomics revealed molecular complexity and diversity of the biological and pathological processes in different tissues following LCMV-mediated infection. We have highlighted various pathways of LCMV-mediated inflammation in the *in vivo* LCMV infection model. The results indicate that cellular responses to LCMV act in a tissue- and time-dependent manner. In the immune-privileged tissue like the neuroretina, there was initially a strong cellular functional inhibition that was temporarily improved but degenerative processes reappeared later. In the kidney, a strong immune response was detected throughout the period with initial inhibition of the mitochondrial function and slight increased degeneration throughout the period. In the spleen, the immune response was weak at 1 week but increased later. Integrin signaling was low at 1 week and increased at 28 weeks. No major degeneration was observed in the spleen. Our results suggest that various identified proteins and pathways may provide clues aiming to unravel complex biological mechanisms and provide a resource for improving our understanding of the molecular basis of LCMV-mediated retinal degeneration. Importantly, the results need to be validated by appropriate functional techniques.

## Data availability statement

The datasets presented in this study can be found in online repositories. The names of the repository/repositories and accession number(s) can be found below: PXD050187 (PRIDE).

## Ethics statement

The animal study was approved by Danish national animal ethics committee (Licence: 2020-15-0201-00586). The study was conducted in accordance with the local legislation and institutional requirements.

## Author contributions

AK: Writing – original draft, Writing – review & editing, Data curation, Formal analysis, Investigation, Methodology, Supervision, Validation, Visualization. MS: Writing – review & editing, Conceptualization, Data curation, Investigation, Methodology, Resources, Supervision. EP: Writing – review & editing, Resources. AA: Writing – review & editing, Investigation. TS: Writing – review & editing, Conceptualization, Funding acquisition, Project administration. HV: Writing – review & editing, Conceptualization, Funding acquisition, Project administration. MN: Writing – review & editing, Conceptualization, Funding acquisition, Project administration, Supervision. BH: Writing – original draft, Writing – review & editing, Conceptualization, Data curation, Formal analysis, Funding acquisition, Investigation, Methodology, Project administration, Resources, Supervision, Validation, Visualization.

## Supplementary material

Supplementary Figure 1 Targeted proteomics using selected reaction monitoring. Generally, the results obtained by discovery-based proteomics were confirmed by selected reaction monitoring (SRM). Blue indicates Log_2_ fold changes observed by discovery-based proteomics and orange the corresponding changes observed with targeted single reaction monitoring (SRM). Generally, SRM confirmed the observation by the discovery method.

Supplementary Figure 2 Ingenuity Pathway Analysis summary of the most significant biological changes at various times in each organ.

Supplementary Figure 3 Antibody response and complement activation in neuroretina and RPE/choroid. Identified proteins involved in the functions “Antibody response” **(A)** and “Complement activation” **(B)** in neuroretina and RPE/choroid. **(A)** In neuroretina a significant increased antibody response could be detected at 8 weeks of infection (z≥2). In RPE/choroid there were significant increased antibody responses at 1 week and at 28 weeks of infection with more proteins involved than in neuroretina. **(B)** Significant perturbation of the function complement activation was seen from 8 weeks in retina and from 1 week in RPE/choroid although the function was not significantly stimulated (z<2). Generally, more proteins were involved in RPE/choroid than in retina.

Supplementary Table 1
**(A)** Retina, 1 week, Identified and differentially expressed proteins. **(B)**. Retina, 8 weeks, Identified and differentially expressed proteins. **(C)**. Retina, 28 weeks, Identified and differentially expressed proteins.

Supplementary Table 2
**(A)** RPE/choroid, 1 week, Identified and differentially expressed proteins. **(B)** RPE/choroid, 8 weeks, Identified and differentially expressed proteins. **(C)** RPE/choroid, 28 weeks, Identified and differentially expressed proteins.

Supplementary Table 3
**(A)** Kidney, 1 week, Identified and differentially expressed proteins. **(B)** Kidney, 8 weeks, Identified and differentially expressed proteins. **(C)** Kidney, 28 weeks, Identified and differentially expressed proteins.

Supplementary Table 4
**(A)** Spleen, 1 week, Identified and differentially expressed proteins. **(B)** Spleen, 8 weeks, Identified and differentially expressed proteins. **(C)** Spleen, 28 weeks, Identified and differentially expressed proteins.

Supplementary Table 5
**(A)** Retina, 1 week, Cannonical pathways. **(B)** Retina, 8 weeks, Cannonical pathways. **(C)** Retina, 28 weeks, Cannonical pathways.

Supplementary Table 6
**(A)** RPE/choroid, 1 week, Cannonical pathways. **(B)** RPE/choroid, 8 weeks, Cannonical pathways. **(C)** RPE/choroid, 28 weeks, Cannonical pathways.

Supplementary Table 7
**(A)** Kidney, 1 week, Cannonical pathways. **(B)** Kidney, 8 weeks, Cannonical pathways. **(C)** Kidney, 28 weeks, Cannonical pathways.

Supplementary Table 8
**(A)** Spleen, 1 week, Cannonical pathways. **(B)** Spleen, 8 weeks, Cannonical pathways. **(C)** Spleen, 28 weeks, Cannonical pathways.

Supplementary Table 9
**(A)** Retina, 1 week, Diseases & Functions. **(B)** Retina, 8 weeks, Diseases & Functions. **(C)** Retina, 28 weeks, Diseases & Functions.

Supplementary Table 10
**(A)** RPE/choroid, 1 week, Diseases & Functions. **(B)** RPE/choroid, 8 weeks, Diseases & Functions. **(C)** RPE/choroid, 28 weeks, Diseases & Functions.

Supplementary Table 11
**(A)** Kidney, 1 week, Diseases & Functions. **(B)** Kidney, 8 weeks, Diseases & Functions. **(C)** Kidney, 28 weeks, Diseases & Functions.

Supplementary Table 12
**(A)** Spleen, 1 week, Diseases & Functions. **(B)** Spleen, 8 weeks, Diseases & Functions. **(C)** Spleen, 28 weeks, Diseases & Functions.
