# Supplementary figures and images for: Neuroretinal degeneration in a mouse model of systemic chronic immune activation observed by proteomics
Source: Front Immunol. 2024 Apr 11;15:1374617. doi: 10.3389/fimmu.2024.1374617 (PMC11043527; doi:10.3389/fimmu.2024.1374617)

Supplementary Fig. S1

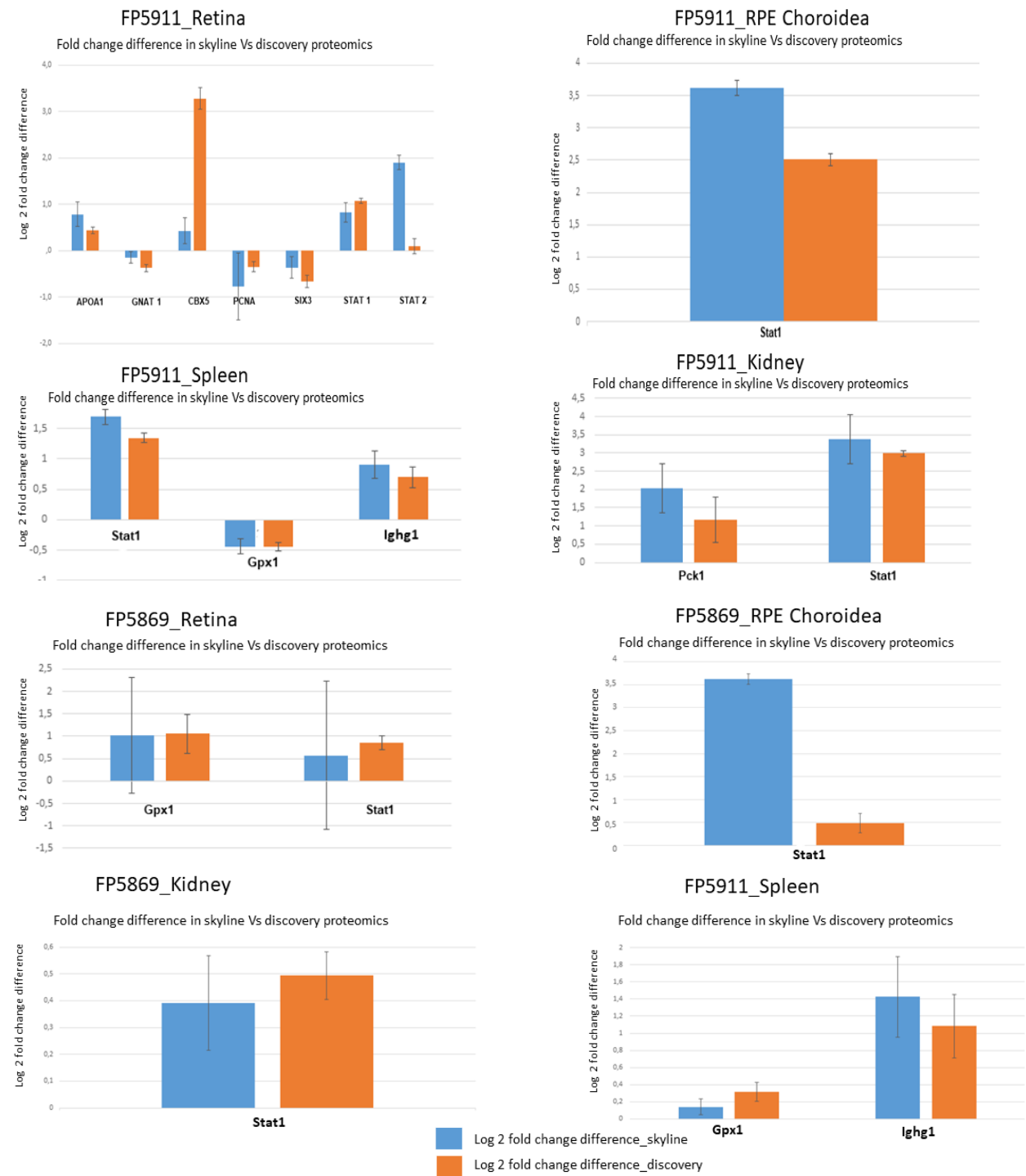

Supplement: Supplementary Figure 1 — Targeted proteomics using selected reaction monitoring. Generally, the results obtained by discovery-based proteomics were confirmed by selected reaction monitoring (SRM). Blue indicates Log2 fold changes observed by discovery-based proteomics and orange the corresponding changes observed with targeted single reaction monitoring (SRM). Generally, SRM confirmed the observation by the discovery method. [file Image_1.pdf]
