# Supplementary material for: Neuroretinal degeneration in a mouse model of systemic chronic immune activation observed by proteomics
Source: Front Immunol. 2024 Apr 11;15:1374617. doi: 10.3389/fimmu.2024.1374617 (PMC11043527; doi:10.3389/fimmu.2024.1374617)
Supplement: Supplementary Figure 2 — Ingenuity Pathway Analysis summary of the most significant biological changes at various times in each organ. [file Image_2.pdf]

# Supplementary Fig. S2A

## Neuroretina

### 1 week

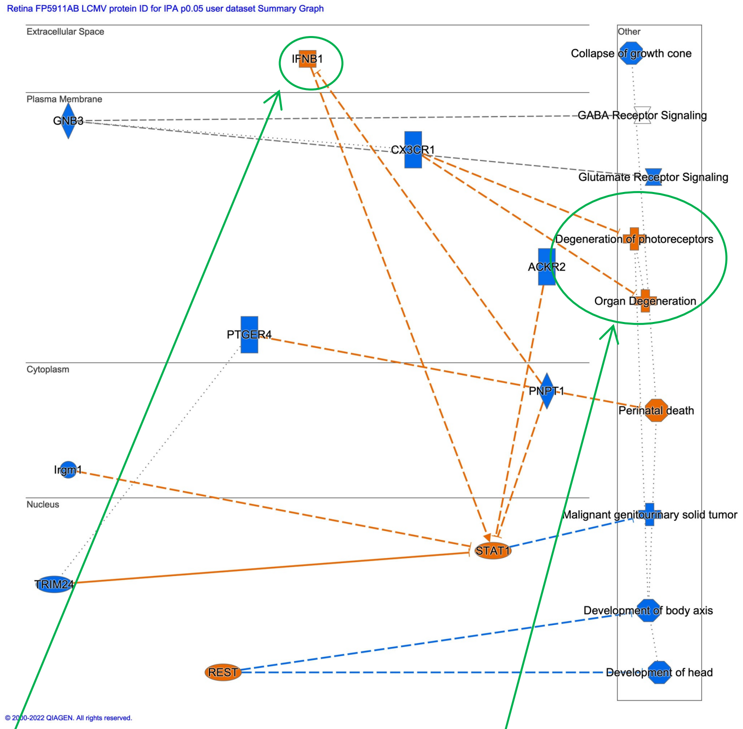


<u>Weak cytokine response (17 protein changes)</u>

No/little immune response

No changed susceptibility to infection

Down: Cell cycle control of DNA replication

Down: Transcription

<u>Substantial degeneration</u>

### 8 weeks

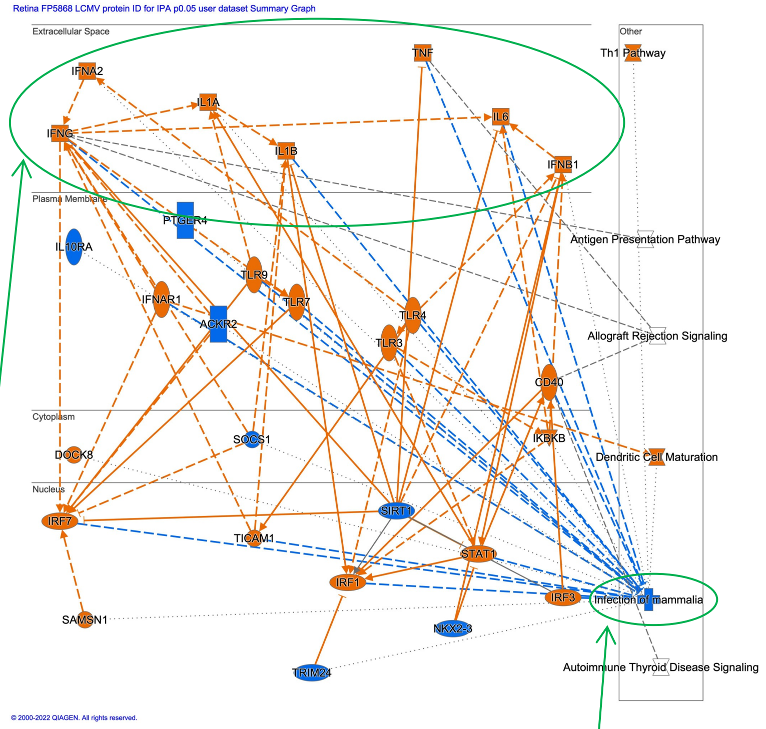


<u>Moderate cytokine response (112 protein changes)</u>

Strong immune response

<u>Decreased susceptibility to infection</u>

No apparent sign of degeneration

### 28 weeks

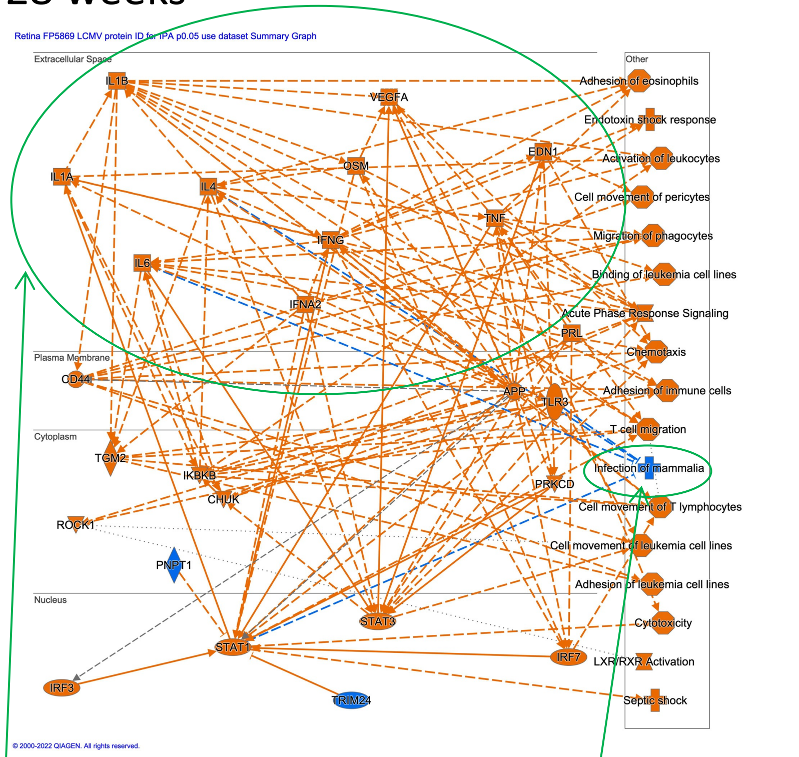


<u>Moderate cytokine response (122 protein changes)</u>

Strong immune response

<u>Decreased susceptibility to infection</u>

Degeneration
