# Supplementary material for: Neuroretinal degeneration in a mouse model of systemic chronic immune activation observed by proteomics
Source: Front Immunol. 2024 Apr 11;15:1374617. doi: 10.3389/fimmu.2024.1374617 (PMC11043527; doi:10.3389/fimmu.2024.1374617)
Supplement: Supplementary Figure 3 — Antibody response and complement activation in neuroretina and RPE/choroid. Identified proteins involved in the functions “Antibody response” (A) and “Complement activation” (B) in neuroretina and RPE/choroid. (A) In neuroretina a significant increased antibody response could be detected at 8 weeks of infection (z≥2). In RPE/choroid there were significant increased antibody responses at 1 week and at 28 weeks of infection with more proteins involved than in neuroretina. (B) Significant perturbation of the function complement activation was seen from 8 weeks in retina and from 1 week in RPE/choroid although the function was not significantly stimulated (z<2). Generally, more proteins were involved in RPE/choroid than in retina. [file Image_3.pdf]

Supplementary Fig. S2B

RPE/choroid
1 week

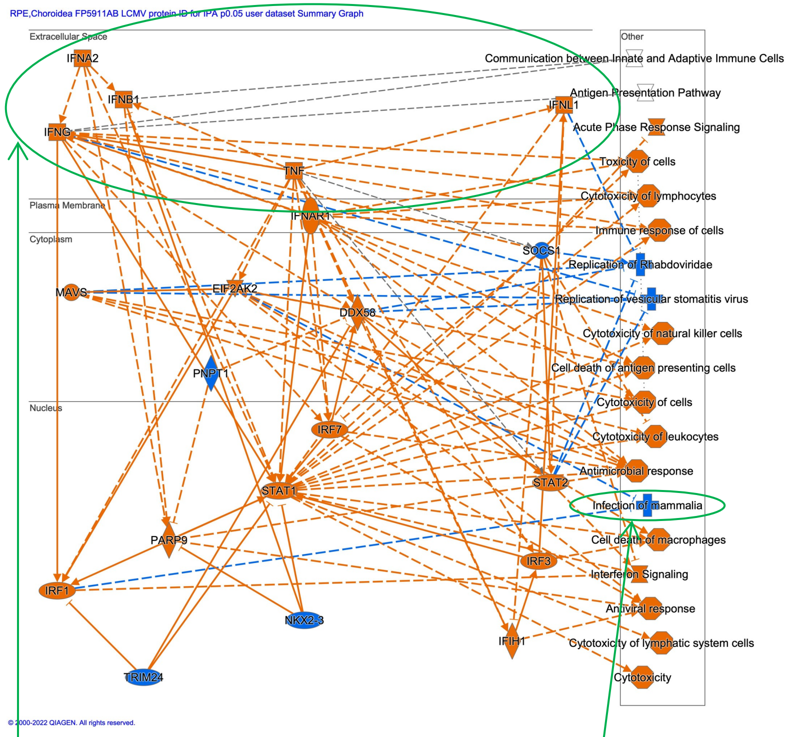

Strong cytokine response (356 protein changes)
Strong immune response
Decreased susceptibility to infection
Up: Cell cycle control of DNA replication
Down: Glycolysis
Up: Actin cytoskelleton signalling
No degeneration

8 weeks

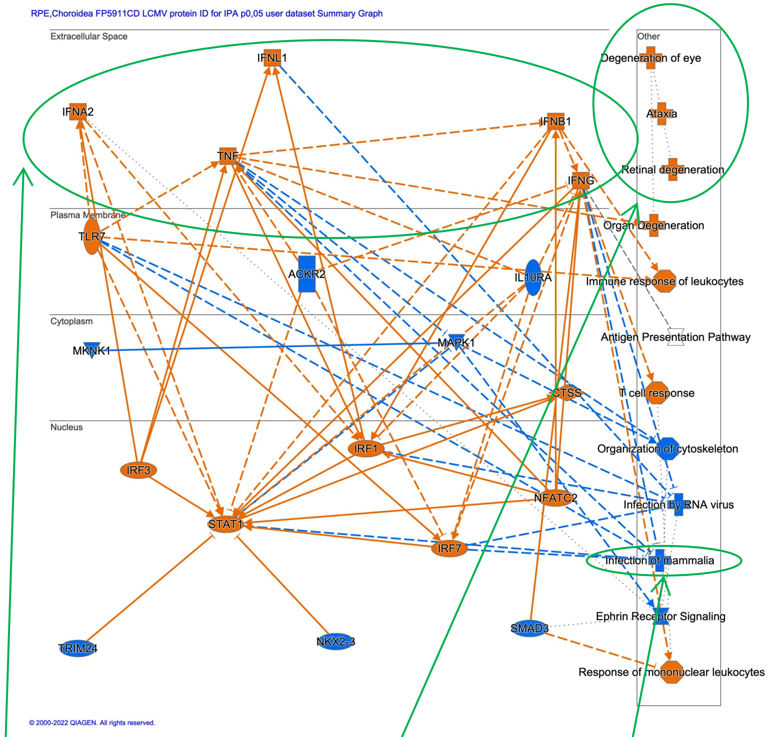

Strong cytokine response (313 protein changes)
Strong immune response
Decreased susceptibility to infection
Down: Integrin signalling

Down: Actin cytoskelleton signalling
Degeneration

28 weeks

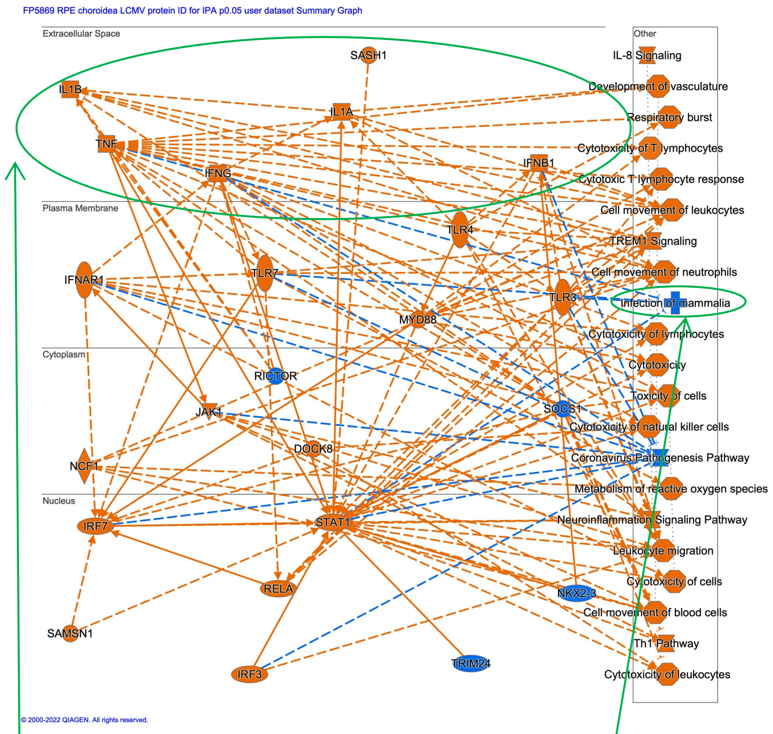

Strong cytokine response (385 protein changes)
Strong immune response
Decreased susceptibility to infection

Up: Actin cytoskelleton signalling
Some degeneration
