# Supplementary material for: Neuroretinal degeneration in a mouse model of systemic chronic immune activation observed by proteomics
Source: Front Immunol. 2024 Apr 11;15:1374617. doi: 10.3389/fimmu.2024.1374617 (PMC11043527; doi:10.3389/fimmu.2024.1374617)
Supplement: Supplementary file 5 [file DataSheet_2.pdf]

Supplementary material

Neuroretinal degeneration in a mouse model of systemic chronic immune activation observed by proteomics

Asif Manzoor Khan, Maria Abildgaard Steffensen, Egle Paskeviciute, Ahmed Basim Abduljabar, Torben Lykke Sørensen, Henrik Vorum, Mogens Holst Nissen and Bent Honoré

Front. Immunology 2024, 15, 1374617

Description of the bioinformatic analysis using the Ingenuity Pathway Analysis of each of the different tissues

Overall biological changes in each tissue at different times

First, we will briefly describe the overall changes seen and subsequently elaborate each tissue in more detail with pathways that are significantly enriched and if possible whether the pathways are stimulated or inhibited. Generally, the effect of LCMV infection in various organs was massive with complex proteins changes, but there were notable differences in the protein response in the different tissues.

In short, the major changes in each tissue were as follows. In the retina, early in the infection period, we observed a weak response of proteins known to be affected by cytokines with no detectable immune response, no detectable changes in the susceptibility to infection and massive degeneration processes (Supplementary Fig. S2A) while later, at 8 weeks, we observed a stronger response of proteins known to be affected by cytokines and a strong immune response with a decrease in the susceptibility to infection and an improvement of degenerative processes while later a more substantial degeneration was found (Supplementary Fig. S2A). The response in RPE-choroid was different from the retina as we detected a strong response of proteins known to be affected by cytokines in the whole infection period with a strong immune response and decrease in the susceptibility to infection from the initial period and throughout. Substantial degeneration was observed from 8 weeks of infection and throughout the period (Supplementary Fig. S2B). In the kidney, we observed a strong response of proteins known to be affected by cytokines, especially in the initial period and then fading out to the later period. The immune response was strong during the whole period with a substantial decrease in the susceptibility to infection, especially at the beginning to the middle of the period (Supplementary Fig. S2C). In the spleen, we observed a moderate response of proteins known to be affected by cytokines in the beginning and mid part of the period while later no protein response to cytokines could be seen but with an increasing growth factor response. The immune response was weak at the beginning and higher in the mid to late period with a decrease in the susceptibility to infection in the mid period (Supplementary Fig. S2D).

Details of biological changes in each tissue at different times

Next, we elaborated the major molecular and functional changes in each organ at different times in more detail. With IPA analysis we obtained information about whether a given set of differentially expressed proteins analysed were significantly enriched for a given pathway, disease or function. Also, there may be enough information to conclude whether a given

pathway, disease or function is significantly stimulated or inhibited. A process with a z value ≥2 was significantly increased while a z value ≤-2 indicates that the process was significantly decreased. In case there was no information about the direction of the perturbed pathway, disease or function, etc., the z value has a question mark, ?.

Retina at 1 week of infection. In retina, at 1 week of infection only a few changes in proteins known to be affected by cytokines were seen. Only 17 observed protein changes could be explained by changes related to one cytokine, IFNB1 (z>2) (Supplementary Fig. S2A).

It was not possible to detect a significant immune response after 1 week of infection. The function "Antibody response" was not significantly perturbed (Supplementary Fig. S3A). This was also the case for the function "Complement activation" (Supplementary Fig. S3B) and the Pathway "Complement system". Also, we couldn't detect a change in the susceptibility to infection.

Canonical pathways connected with DNA replication and repair as well as transcription were perturbed (Supplementary Table S5A). Significant inhibition was seen with the pathways "Cell cycle control of chromosomal replication" (z<-2), "Assembly of RNA polymerase II complex" (z<-2) and "Assembly of RNA polymerase III complex" (z<-2). Other pathways were observed to be perturbed, "DNA Double-Strand Break Repair proteins by Homologous Recombination" (z=?) with four proteins downregulated and "Role of BRCA1 in DNA damage response" with a tendency (z=-1.342) to be inhibited (11 proteins down and 1 up).

Several signalling pathways were perturbed and most with a tendency to be inhibited, "Phototransduction pathway" (z=?, 17 proteins down) with similar observations from Cytoscape (detection of light stimulus involved in visual perception, Fig. 4). Other pathways that were perturbed were "GABA receptor signalling" (z=?, 24 proteins down), "TR/RXR activation" (z=?, 13 proteins down, 1 up), "Glutamate receptor signalling" (z=-1.890, 17 proteins down) and "Androgen signaling" (z-score=-1.414, 28 proteins down).

Some metabolic pathways concerning lipids and amino acids were significantly inhibited, "Triacylglycerol biosynthesis" (z<-2) and "Uptake of amino acids" (z<-2).

Finally, degenerative processes were substantial after 1 week of infection as seen from the disease or function: "Degeneration of photoreceptors" (z>2), "Retinal degeneration" (z>2), "Degeneration of eye" (z>2), and decreased amounts of photoreceptors "Quantity of photoreceptors" (z<-2), "Function of photoreceptors" (z<-2), "Response of photoreceptors" (z<-2). Table 2 (and Supplementary Table S9A).

Retina at 8 weeks of infection. At 8 weeks of infection a moderate change in proteins known to be affected by cytokines were found with changes in around 112 proteins that could be explained by changes to 18 cytokines (Supplementary Fig. S2A). With the canonical pathways, we observed a significant stimulated interferon signalling, "Interferon signalling" (z>2) and also a tendency to increased IL-3 (z=0.447), IL-6 (z=0.447), IL-7 (z=1) and IL-8 (z=1.667) signalling.

As opposed to 1 week of infection at 8 weeks we detected a strong immune response (Supplementary Fig. S3A) with several functions significantly stimulated such as "Immune response of cells" (z>2), "Leukocyte migration" (z>2), "Migration of phagocytes" (z>2), "Th1 pathway" (z>2) and the function "Antibody response" (z>2) (Supplementary Table S9B). Another significantly perturbed canonical pathway was "Antigen presentation pathway" with 9 proteins up including the proteasome subunits PSMB8 and PSMB9 (z=?). The pathway "Complement system" (z=?) was perturbed with 3 proteins upregulated, C1QC, C4A/C4B,

ITGB2 (Supplementary Table S5B) and the function “Complement activation” (z=?) was perturbed (Supplementary Fig. S3B).

A strong decrease in the susceptibility to infection was found at 8 weeks as opposed to 1 week, “Infection of mammalia” (z<-2) (Supplementary Table S9B).

“Ferroptosis signalling pathway” was perturbed with a tendency to be increased (z=1.633) (Supplementary Table S5B).

Degenerative processes specifically connected with retinal visual functions could not be detected (Fig. 4). Only unspecific processes were detected to a slight extend, such as increased cell death of epithelial cells “Cell death of epithelial cells” (z>2) (Supplementary Table S9B).

Retina at 28 weeks of infection. Around 122 protein changes could be explained by changes of 18 cytokines (Supplementary Fig. S2A).

A strong immune response was observed as seen from “Inflammatory response” (z>2) and with recruitment of immune cells, “Recruitment of leukocytes” (z>2), “Lymphocyte migration” (z>2), “Leukocyte extravasation signalling” (z>2) and “Neuroinflammatory signalling pathway” (z>2) (Supplementary Table S9C). The function “Antibody response” was not significantly perturbed (Supplementary Fig. S3A) but the function “Complement activation” (z=?) was significantly perturbed (Supplementary Fig. S3B). Also, the pathway “Complement system” (z=?) was significantly perturbed with 1 complement protein upregulated, C3.

A strong decrease in the susceptibility to infection was found, “Infection of mammalia” (z<-2) (Supplementary Table S9C).

Several signalling pathways were stimulated such as “LXR/RXR activation” (z>2) and “Tec kinase signalling” (z>2). The pathway “Iron homeostasis signalling pathway” was perturbed with 6 proteins up and 1 down (z=?) (Supplementary Table S5C).

Degenerative processes in retina were apparent from the diseases and functions: “Retinal degeneration” (z>2), “Degeneration of eye” (z>2) and “Degeneration of photoreceptors” (z>2) that were stimulated. There was a significant decreased “Quantity of photoreceptors” (z<-2). Also, the pathway “Phototransduction pathway” was perturbed with 12 proteins down (z=?) and the function was thereby presumably inhibited (Supplementary Table S9C). The Cytoscape analysis also confirmed the decreased function of retina, Fig. 4.

RPE-choroid at 1 week of infection. Changes in about 356 proteins could be explained by changes of 35 cytokines and also the pathway “Interferon signalling” (z>2) was stimulated (Supplementary Fig. S2B).

The immune response was stimulated as seen from the stimulation of several pathways and functions (Supplementary Tables S6A and S10A), such as “Acute phase response signalling” (z>2), “Proliferation of lymphocytes” (z>2), “Leukocyte migration” (z>2), “Immune response of leukocytes” (z>2) as well as from the stimulation of signalling pathways: “CD28 signalling in T helper cells” (z>2) and “Neuroinflammation signalling pathway” (z>2) and the function “Antibody response” (z>2) (Supplementary Fig. S3A). Despite of increased cellular infiltration of immune cells and increased proliferation, the data also indicated increased cell death, “Cell death of immune cells” (z>2). The pathway “Complement system” (z=0.333) was perturbed with 10 proteins upregulated. The function “Complement activation” (z=0.225) was also perturbed (Supplementary Fig. S3B) although not significantly stimulated.

We found a strong decrease in the susceptibility to infection "Infection of mammalia" (z<-2) (Supplementary Table S10A).

As opposed to retina at 1 week we found that control with DNA replication was stimulated in RPE-choroid "Cell cycle control of chromosomal replication" (z>2) (Supplementary Table S6A).

Several signalling pathways were stimulated, "CDC42 signalling" (z>2), "TREM1 signalling" (z>2), "Ephrin receptor signalling" (z>2), RAC signalling (z>2) and "Actin cytoskeleton signalling" (z>2) (Supplementary Table S6A).

Metabolically, we found that glycolysis was inhibited, "Glycolysis I" (z<-2) (Supplementary Table S6A).

Degeneration processes of the RPE-choroid was not detected in the early phase of infection (Supplementary Fig. S2B).

RPE-choroid at 8 weeks of infection. Changes in about 313 proteins could be explained by changes of 21 cytokines (Supplementary Fig. S2B) and also the pathway "Interferon signalling" (z>2) was stimulated (Supplementary Fig. S6B.)

There was a stimulated immune response "Immune response of leukocytes" (z>2) and "Recruitment of leukocytes" (z>2) and the pathway "Neuroinflammation signalling pathway" showed a strong tendency (z=1.964) to be stimulated. The function "Antibody response" was not perturbed (Fig. S3A). The pathway "Complement system" (z=0.707) was perturbed with 9 proteins upregulated and the function "Complement activation" was also perturbed (Supplementary Fig. S3B).

A strong decrease in the susceptibility to infection was seen, "Infection of mammalia" (z<-2) (Supplementary Table S10B).

Several signalling pathways were observed to be inhibited, "Integrin signalling" (z<-2), "Actin cytoskeleton signalling" (z<-2) and "Ephrin receptor signalling" (z<-2) while "Neuregulin signalling" showed a tendency (z=-1.414) to be inhibited. A few pathways were stimulated, including "RHOGDI signalling" (z>2) (Supplementary Table S6B).

Several degenerative functions were observed including "Degeneration of eye" (z>2), "Retinal degeneration" (z>2) and "Degeneration of photoreceptors" (z>2) (Supplementary Table S10B). In the "Phototransduction pathway" (z=?) we found 19 proteins downregulated, so the pathway was presumably inhibited (Supplementary Table S6B). Reduced phototransduction was also confirmed by Cytoscape (Fig. 4).

RPE-choroid at 28 weeks of infection. Changes in about 385 proteins could be explained by changes of 29 cytokines (Supplementary Fig. S2B) and also several cytokine signalling pathways were stimulated (z>2) "IL-3", "IL-7", "IL-8", "IL-9", "IL-15" as well as "IL-22 signalling" (Supplementary Table S6C).

There was a strong immune response, "Inflammatory response" (z>2), "Immune response of leukocytes" (z>2), "Antibody response" (z>2) (Supplementary Fig. S3A) with proliferation of immune cells, "Proliferation of immune cells" (z>2) (Supplementary Table S10C). The stimulation of naïve T cells into helper cells was also increased as seen from, "Th1 pathway" (z>2) and "Th2 pathway" (z>2). Also, the acute phase response was stimulated "Acute phase response signalling" (z>2) (Supplementary Table S6C) and the complement system showed a tendency to be stimulated "Complement system (activation) (z=1.508) with 14 proteins upregulated (Supplementary Table S6C) and the function "Complement activation" was

perturbed and showed a tendency to be stimulated (z=1.803) ((Supplementary Table S10C and Supplementary Fig. S3B).

We observed a strong decrease in the susceptibility to infection “Infection of mammalia” (z<-2) (Supplementary Table S10C).

Several signal transduction pathways were mainly stimulated, e.g., “EIF2 signalling” (z>2), “Phospholipase C signalling” (z>2), “Actin cytoskeleton signalling” (z>2), “HMGB1 signalling” (z>2), TGF-β signalling (z>2), “Nuclear factor-κB” (NF-κB) activation by viruses” (z>2), “mTOR signalling” (z>2), “VEGF family ligand-receptor interactions” (z>2), “Inhibition of angiogenesis by TSP-1” (z>2) and “Endoplasminc reticulum stress pathway” (z>2) (Supplementary Table S6C).

At 28 weeks the set of proteins changed were significantly associated with degeneration, “Macular degeneration” (z=?) although the direction of the change could not be significantly specified (Supplementary Table S10C). Also, in the pathway “Phototransduction pathway” (z=?) we found 12 proteins downregulated indicating that it may be inhibited (Supplementary Table S6C). Decreased function and degeneration was also confirmed by Cytoscape (Fig. 4).

Kidney at 1 week of infection. Changes in about 497 proteins could be explained by changes of 28 cytokines (Supplementary Fig. S2C) and the interferon signalling pathway was stimulated, “Interferon signalling” (z>2) (Supplementary Table S7A).

There is a strong immune response with stimulation of “Immune response of leukocytes” (z>2) and with an increased recruitment of monocytes (z>2), phagocytes (z>2), leukocytes (z>2) and granulocytes (z>2) (Supplementary Table S11A). “Systemic lupus erythematosus in B cell signalling pathway” (z>2) was also stimulated (Supplementary Table S7A). Although there was proliferation there was also an increased cell death of immune cells “Cell death of immune cells” (z>2) and “Cell death of antigen presenting cells” (z>2) (Supplementary Table S11A).

The susceptibility to infection was decreased as seen from the inhibition of “Infection of mammalia” (z<-2) (Supplementary Table S11A).

A few signalling pathways were stimulated “Sirtulin signalling pathway” (z>2), while others were significantly perturbed “Gucocorticoid receptor signalling” (z=?) (57 proteins down, 29 up), “FXR/RXR activation“ (z=?) (17 proteins up, 13 down) with no clear direction (Supplementary Table S7A).

Metabolically, the most significant changes were a strong downregulation of 65 proteins in oxidative phosphorylation, “Oxidative phosphorylation” (z<-2) and with a presumed dysfunction of the mitochondrion, “Mitochondrial dysfunction” (z=?, 85 proteins down of 88). Also, the Acetyl-CoA biosynthesis and the TCA cycle were inhibited “Acetyl-CoA biosynthesis I (Pyruvate dehydrogenase complex)” (z<-2) and “TCA cycle II” (z<-2). Several metabolic pathways of amino acid degradation were downregulated (z<-2) such as observed with the amino acids valine, leucine, isoleucine, tryptophan and phenylalanine. Also, cholesterol biosynthesis was perturbed with a tendency to be inhibited (z<-0.905) (Supplementary Table S7A).

Some stress response pathways were stimulated “NRF2-mediated oxidative stress response” (z>2) (Supplementary Table S7A).

Apparently, degeneration is not a great problem and actually with *inhibition* of impairment processes, “Renal impairment” (z<-2) and “Failure of kidney” (z<-2) (Supplementary Table S11A).

Kidney at 8 weeks of infection. Changes in about 117 proteins could be explained by changes of 18 cytokines (Supplementary Fig. S2C) and the interferon signalling pathway was stimulated, “Interferon signalling” (z>2) (Supplementary Table S7B).

There was a stimulation of the immune response as found from “Immune response of cells” (z>2), “Quantity of lymphocytes” (z>2), “Proliferation of immune cells” (z>2) (Supplementary Table S11B), “Dendridic cell maturation” (z>2), “Th1 pathway” (z>2) and “Role of hypercytokinemia/hyperchemokinemia in the pathogenesis of influenza” (z>2) (Supplementary Table S7B). Some pathways were perturbed and might be stimulated “Antigen presentation pathway” (z=?) with 8 proteins up (Supplementary Table S7B). Also, we found an increased cytolysis of immune cells “Cytolysis of leukocytes” (z>2) (Supplementary Table S11B).

The susceptibility to infection was decreased “Infection of mammalia” (z<-2) (Supplementary Table S11B).

A few metabolic changes were seen with amino acids, “Valine degradation I” (z=-2) (Supplementary Table S7B).

Degeneration was only a problem to a little extend “Renal impairment” ($p=2.96x10^{-3}$, z=?) (Supplementary Table S11B).

Kidney at 28 weeks of infection. Changes in about 86 proteins could be explained by changes of 6 cytokines (Supplementary Fig. S2C).

The transcription regulator STAT1 apparently plays no major role late in the kidney infection. This is opposed to the central effect of STAT1 in the kidney in the early- and mid-period as well as in the whole period in retina and RPE-choroid (Supplementary Fig. S2C).

The immune response is present as seen from the stimulated pathways “Dendritic cell maturation” (z>2), “PI3K signalling in B lymphocytes” (z>2), “CD28 signalling in T helper cells” (z>2), “Role of NFAT in regulation of the immune response” (z>2) (Supplementary Table S7C) and from the function: “Immune response of cells” (z>2) (Supplementary Table S11C).

Other pathways like chemokine signalling (z=0.816) with 5 proteins up and 1 down and “Phospholipase C signalling” also tended (z=1.508) to be stimulated (Supplementary Table S7C).

The susceptibility to infection was decreased, “Infection of mammalia” (z<-2) (Supplementary Table S11C).

Degeneration may be a problem to some extent, “End stage renal disease” ($p=7.56x10^{-3}$, z=?) (Supplementary Table S11C).

Spleen at 1 week of infection. The changed expression of 487 proteins could be explained by changes of 11 cytokines (Supplementary Fig. S2D) and we also observed stimulation of the pathway, “Role of hypercytokinemia/hyperchemokinemia in the pathogenesis of influenza” (z>2) (Supplementary Table S8A).

The immune response was not strong as we found a decreased quantity of immune cells, “Quantity of lymphoid cells” (z<-2) (Supplementary Table S12A). Also, a number of signalling pathways in the immune response was inhibited, ”PI3K signalling in B lymphocytes” (z<-2), “B cell receptor signalling” (z<-2), “T cell receptor signalling” (z<-2) and “CD28 signalling in T helper cells” (z<-2) (Supplementary Table S8A). Finally, we observed a stimulation of the inflammation process “Inflammation of body cavity” (z>2) (Supplementary Table S12A).

There is a decreased susceptibility to infection “Infection by coronavirus” (z<-2) (Supplementary Table S12A).

The control of DNA replication was stimulated “Cell cycle control of chromosomal replication” (z>2) (Supplementary Table S8A).

With respect to protein synthesis, we found that the initiation was stimulated from “IEF2 signalling” (z>2) and “tRNA charging” (z>2) (Supplementary Table S8A). However, the overall translation process was observed to be inhibited “Translation of mRNA” (z<-2) (Supplementary Table S12A).

The communication between the extracellular matrix and the cell interior was downregulated as the signalling pathway “Integrin signalling” (z<-2) and “Actin cytoskeleton signalling (z<-2) were inhibited (Supplementary Table S8A). Protein metabolism was increased “Metabolism of protein” (z>2) (Supplementary Table S12A). The “BAG2 signalling pathway” was perturbed and tended to be stimulated (z=0.688) (Supplementary Table S8A). “Granzyme B signalling pathway” tended to be downregulated (z=-0.632) (Supplementary Table S8A).

Degradation was not a major observation.

Spleen at 8 weeks of infection. The expression of about 158 proteins could be explained by changes of 12 cytokines (Supplementary Fig. S2D). Also, here we found stimulation of the pathway, “Role of hypercytokinemia/hyperchemokinemia in the pathogenesis of influenza” (z>2) (Supplementary Table S8B).

There was an immune response, “Quantity of leukocytes” (z>2), “Lymphocyte migration” (z>2) and the function “Antibody response” (z>2) (Supplementary Table S12B).

The susceptibility to infection was decreased, “Infection of mammalia” (z<-2) and “Inflammation of organ” (z<-2) (Supplementary Table S12B).

Metabollically, we found stimulation of oxidative phosphorylation, “Oxidative phosphorylation” (z>2) (Supplementary Table S8B).

Some signalling pathways were stimulated “14-3-3-mediated signalling” (z>2) and “ERK5 signalling” (z>2) and “LXR/RXR activation” (z>2) while few were inhibited “HIPPO signalling” (z<-2) (Supplementary Table S8B).

Degradation was not a major problem.

Spleen at 28 weeks of infection. At 28 weeks no protein changes were observed that could be explained by changes in the exposure to cytokines. Instead, around 156 protein changes could be explained by changes of 8 growth factors (Supplementary Fig. S2D).

There was an element of immune response as seen from the function “Stimulation of leukocytes” (z>2) (Supplementary Table S12C). Also, the pathway “Role of IL-17F in allergic inflammatory airway diseases” (z>2) and “Acute phase response signaling” (z>2) were stimulated (Supplementary Table S8C). There was no major detectable effect of the transcription regulator STAT1 as also seen in kidney at the late state of infection.

Transcription was increased “Transcription” (z>2) (Supplementary Table S12C) consistent with a tendency towards a stimulation of the pathway “Assembly of RNA polymerase II complex” (z=1.667) (Supplementary Table S8C).

The communication between cells and the extracellular matrix was stimulated “Integrin signalling” (z>2) and the integrin linked kinase pathway that links integrins to the cytoskeleton also tended to be stimulated “ILK signalling” (z=1.807) (Supplementary Table S8C). The intracellular signalling pathway “Phospholipase C signalling” (z>2) that may be involved in the organization of the cytoskeleton was also stimulated (Supplementary Table S8C). Finally, the intracellular organization and formation of the cytoskeleton were stimulated, “Actin

cytoskeleton signalling” (z>2) (Supplementary Table S8C) and “Formation of cytoskeleton” (z>2) (Supplementary Table S12C).

Degeneration at the late state was not a problem with inhibition of “Growth failure” (z<-2) and “Organismal death” (z<-2) and stimulation of the functions “Quantity of cells” (z>2) and “Formation of filaments” (z>2) further indicated this. On the contrary, cell proliferation and cytoskeletal changes were dominant as “Neoplasia of cells” (z>2) and “Organization of cytoskeleton” (z>2) were stimulated (Supplementary Table S12C).
