# Supplementary material for: Neuroretinal degeneration in a mouse model of systemic chronic immune activation observed by proteomics
Source: Front Immunol. 2024 Apr 11;15:1374617. doi: 10.3389/fimmu.2024.1374617 (PMC11043527; doi:10.3389/fimmu.2024.1374617)
Supplement: Supplementary file 6 [file Image_4.pdf]

Supplementary Fig. S2C

Kidney
1 week

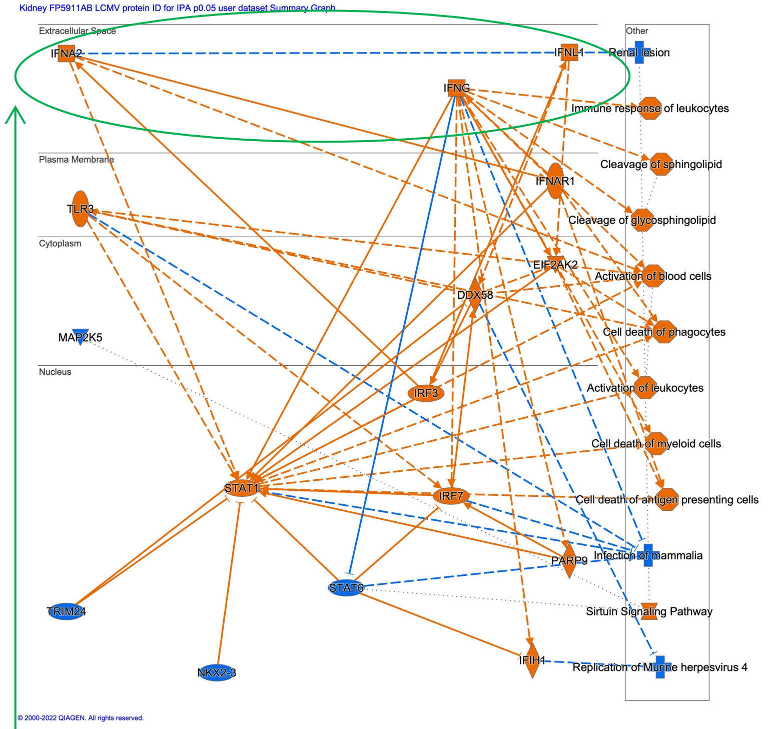

Strong cytokine response (497 protein changes)

Strong immune response

Decreased susceptibility to infection

Down: Acetyl-CoA synthesis

Down: TCA cycle

Down: Oxidative phosphorylation

No degeneration

8 weeks

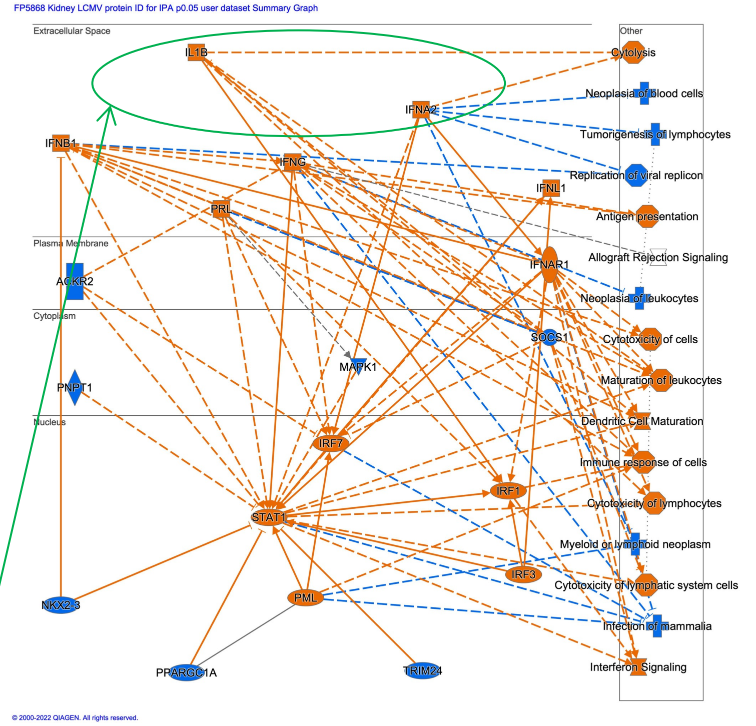

Moderate cytokine response (117 protein changes)

Strong immune response

Decreased susceptibility to infection

Little degeneration

28 weeks

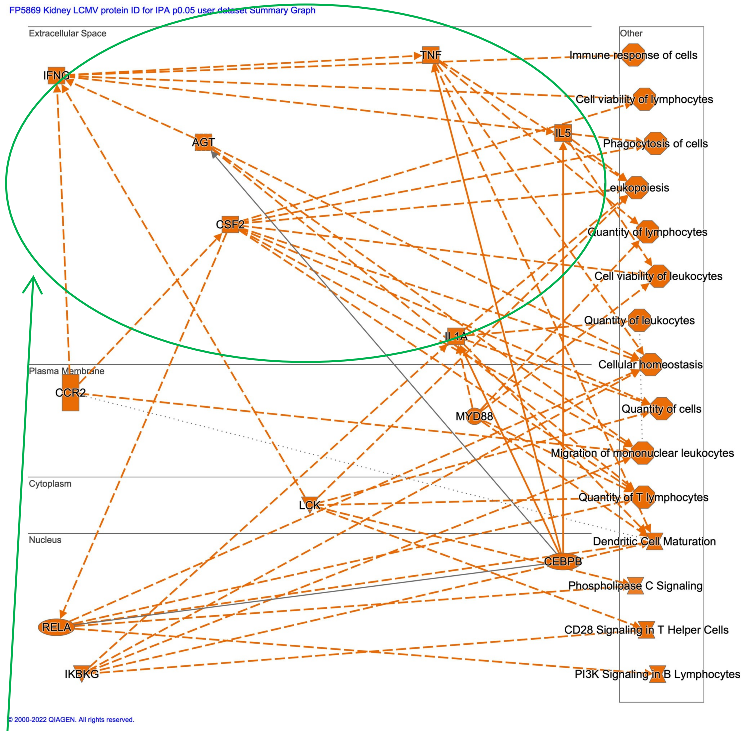

Moderate cytokine response (86 protein changes)

Strong immune response

Decreased susceptibility to infection

No major STAT1 effect

Some degeneration
