# Supplementary material for: Neuroretinal degeneration in a mouse model of systemic chronic immune activation observed by proteomics
Source: Front Immunol. 2024 Apr 11;15:1374617. doi: 10.3389/fimmu.2024.1374617 (PMC11043527; doi:10.3389/fimmu.2024.1374617)
Supplement: Supplementary file 7 [file Image_5.pdf]

Supplementary Fig. S2D

Spleen
1 week

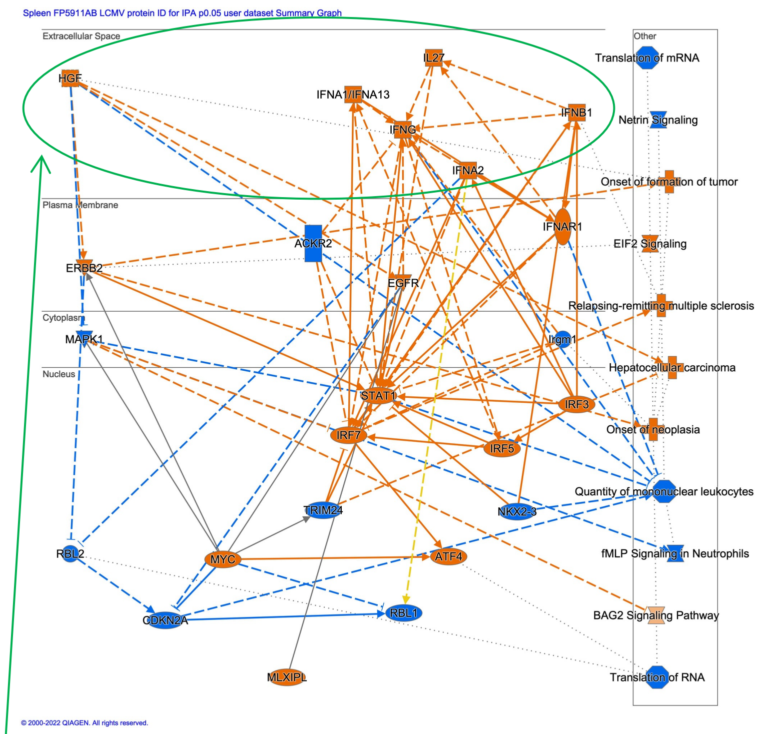

8 weeks

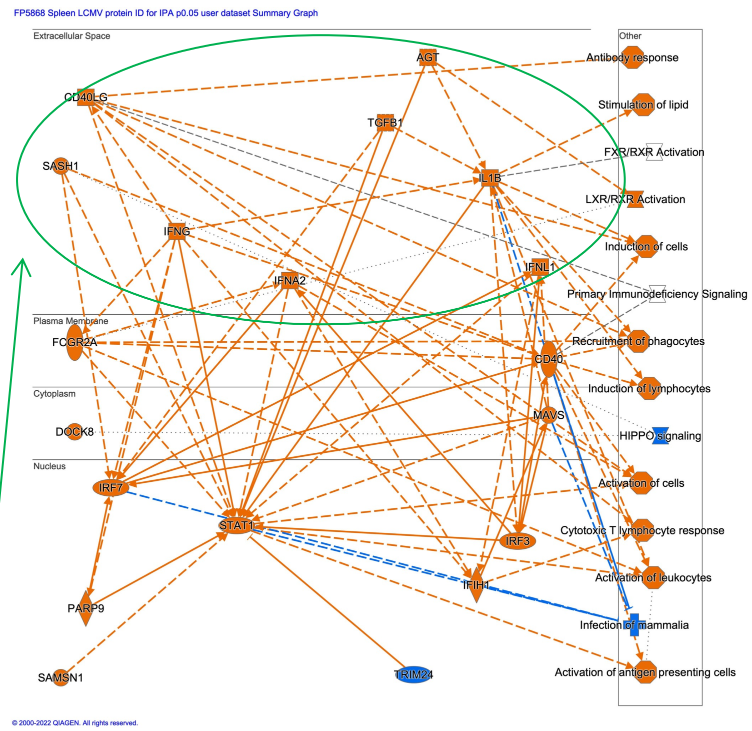

28 weeks

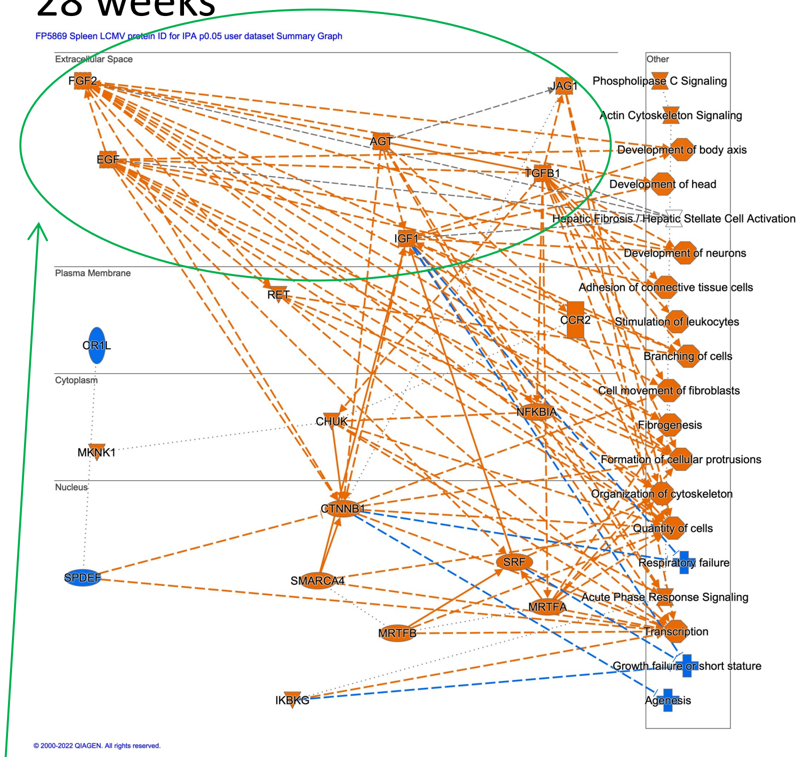

1 week:

<u>Strong cytokine response (487 protein changes)</u>

Weak immune response
Decreased susceptibility to infection
Up: Cell cycle control of DNA replication
Up: IEF2 signalling (trancription initiation)
Down: Translation
Down: Integrin signalling
No degeneration

8 weeks:

<u>Moderate cytokine response (158 protein changes)</u>

Immune response
Decreased susceptibility to infection
Up: Oxidative phosphorylation

No degeneration

28 weeks:

No cytokine response
<u>Growth factor response (156 protein changes)</u>
Immune response
Up: Transcription

Up: Integrin signalling
Up: Formation of cytoskelleton
No major STAT1 effect
No degeneration
