# Supplementary material for: Neuroretinal degeneration in a mouse model of systemic chronic immune activation observed by proteomics
Source: Front Immunol. 2024 Apr 11;15:1374617. doi: 10.3389/fimmu.2024.1374617 (PMC11043527; doi:10.3389/fimmu.2024.1374617)

Fig. S3A “Antibody response”

Neuroretina

1 week

Not Significant

8 weeks

Z=2.412

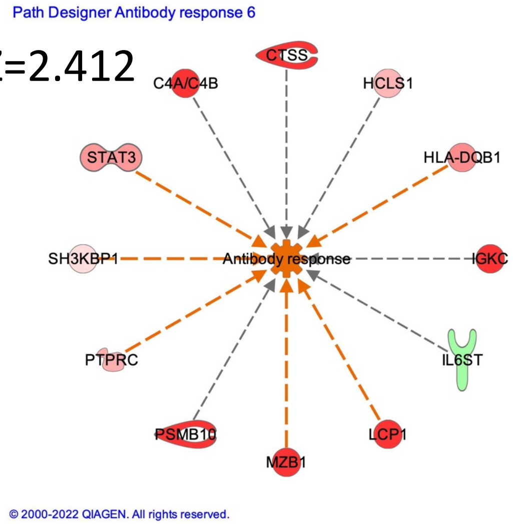

28 weeks

Not Significant

RPE/choroid

1 week

Z=3.138

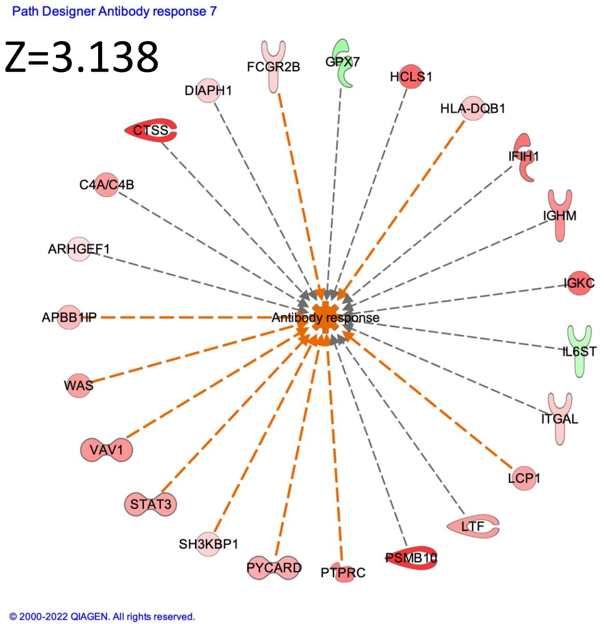

8 weeks

Not Significant

28 weeks

Z=3.138

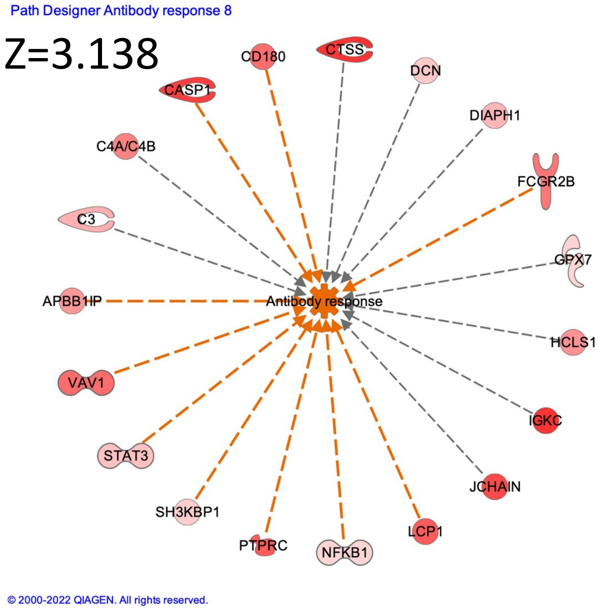

Supplement: Supplementary file 8 [file Image_6.pdf]
