# Supplementary material for: Neuroretinal degeneration in a mouse model of systemic chronic immune activation observed by proteomics
Source: Front Immunol. 2024 Apr 11;15:1374617. doi: 10.3389/fimmu.2024.1374617 (PMC11043527; doi:10.3389/fimmu.2024.1374617)

Fig. S3B “Complement activation”

Neuroretina

1 week

Not Significant

8 weeks

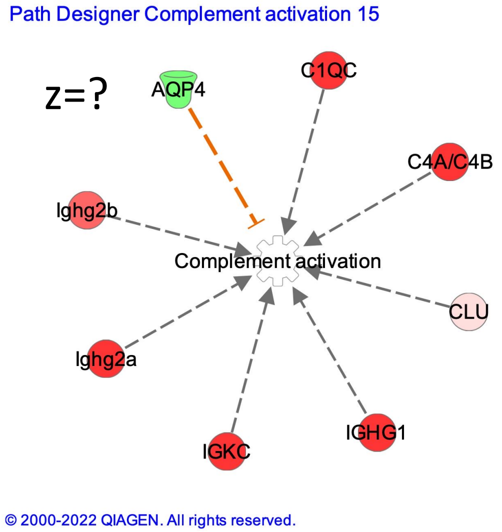

28 weeks

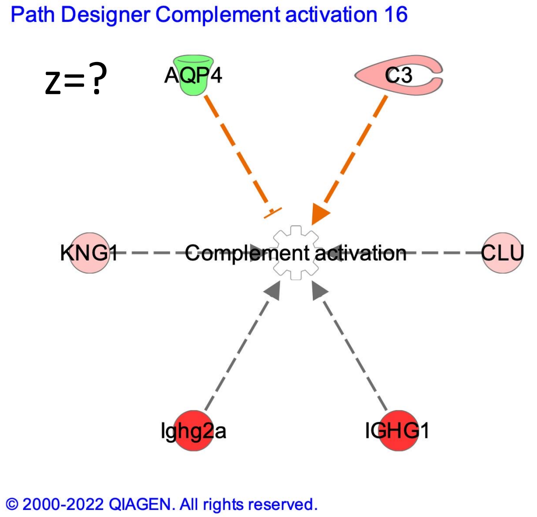

RPE/choroid

1 week

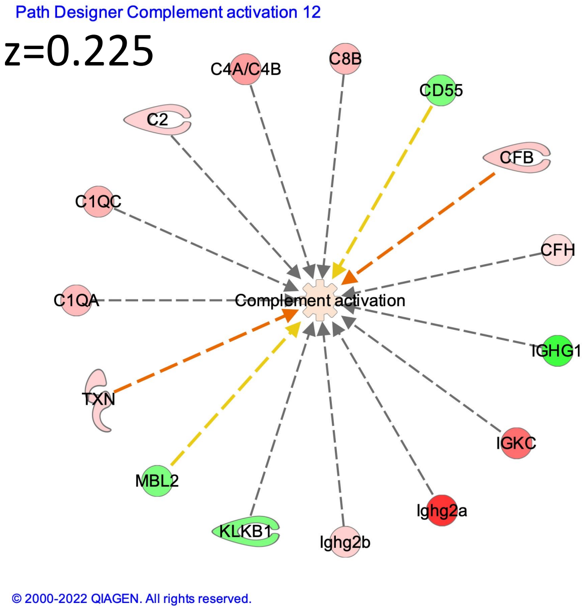

8 weeks

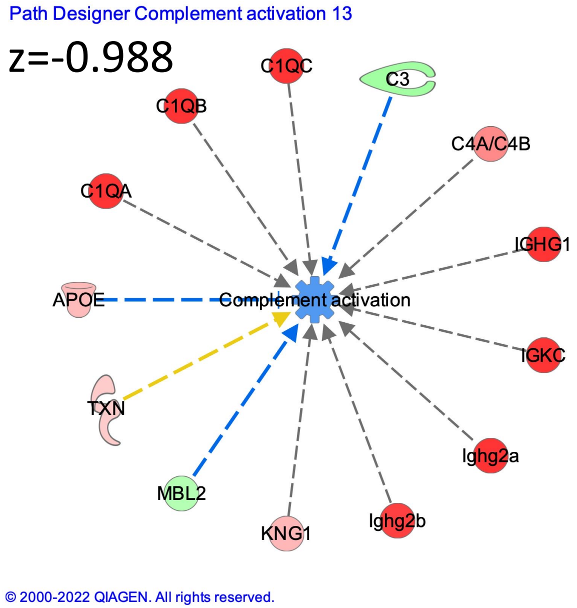

28 weeks

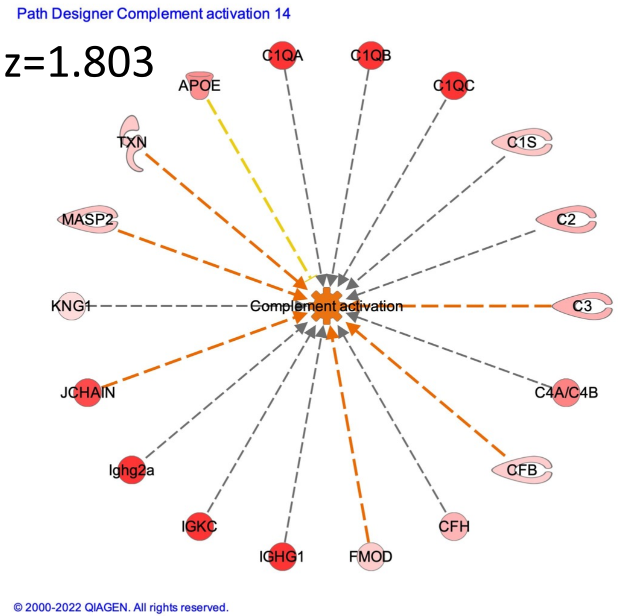

Supplement: Supplementary file 9 [file Image_7.pdf]
